# Genome-wide survey of switchgrass NACs family provides new insights into motif and structure arrangements and reveals stress-related and tissue-specific NACs

**DOI:** 10.1038/s41598-017-03435-z

**Published:** 2017-06-08

**Authors:** Haidong Yan, Ailing Zhang, Yuntian Ye, Bin Xu, Jing Chen, Xiaoyan He, Chengran Wang, Sifan Zhou, Xinquan Zhang, Yan Peng, Xiao Ma, Yanhong Yan, Linkai Huang

**Affiliations:** 1 Department of Grassland Science, Animal Science and Technology College, Sichuan Agricultural University, Chengdu, 611130 China; 2 Department of Horticulture, College of Horticulture, Sichuan Agricultural University, Chengdu, 611130 China; 3 College of Grassland Science, Nanjing Agricultural University, Nanjing, 210095 China

## Abstract

NAC proteins comprise of a plant-specific transcription factor (TF) family and play important roles in plant development and stress responses. Switchgrass (*Panicum virgatum*) is the prime candidate and model bioenergy grass across the world. Excavating agronomically valuable genes is important for switchgrass molecular breeding. In this study, a total of 251 switchgrass NAC (*PvNAC*s) family genes clustered into 19 subgroups were analyzed, and those potentially involved in stress response or tissue-specific expression patterns were pinpointed. Specifically, 27 *PvNAC*s were considered as abiotic stress-related including four membrane-associated ones. Among 40 tissue-specific *PvNAC*s expression patterns eight factors were identified that might be relevant for lignin biosynthesis and/or secondary cell wall formation. Conserved functional domains and motifs were also identified among the PvNACs and potential association between these motifs and their predicted functions were proposed, that might encourage experimental studies to use PvNACs as possible targets to improve biomass production and abiotic stress tolerance.

## Introduction

NAC transcription factors (TFs) are the largest and plant-specific TF family^1, 2^, with the featured NAC domain firstly found in the N-terminal of petunia NAM, *Arabidopsis thaliana* ATAF1/2 and CUC2 and named after those three genes^3–5^. Typically, the conserved N-terminal DNA-binding domains (DBD) of NACs contain about 160 amino acid residues that can be further classified into five subdomains (designated as A-E subdomains), while their C-terminal regions contain the transcriptional activation/repression regions (TARs or TRRs) with highly divergent sequences^3, 6^ that might also be involved in protein-protein interactions and contribute to their regulation specificities^7^. The NACs of *Arabidopsis* and rice (*Oryza sativa*) can be classified into two groups and 18 subgroups according to their primary protein structures (i.e. amino acid sequences)^8^. Within each subgroup, the TARs appear to have conserved motifs corresponding to their NAC domain structures, suggesting that NAC proteins in each subgroup might evolve to have similar functions^8^. This working model proposed by Ooka^8^ has been proven to be valid in many studies using experimental approaches, and could be used as a basis for target gene identification and characterization in switchgrass as well.

NACs play important roles in plant development and response to abiotic stresses. For examples, vascular-related *NAC* genes, such as *VND6*, *VND7*, *SND1*, *NST1*, and *NST3*, were all involved in secondary cell wall thickening in *Arabidopsis* ^9–11^. Overexpression of *NST1* or *NST3* induced ectopic secondary wall thickening, while *nst1*/*nst3* mutants had severely suppressed lignification in the aboveground tissues^9^. Several switchgrass *NACs* (*PvSWNs*) and a *MYB* gene were highly expressed in stems and closely associated with sclerenchyma cells; overexpression of some of these genes in *Arabidopsis snd1*/*nst1* or *mby46*/*myb83* double mutant rescued the secondary wall defects by activating of the biosynthetic genes for cellulose, lignin and xylan and ectopic deposition of secondary walls in parenchymatous cells^12^. Many reported *NAC* family genes were involved in plant abiotic and biotic stress tolerances and the regulation of leaf senescence^13, 14^. For examples, the abiotic stress-inducible *NAC* genes, *ATAF1* acted as a negative regulator in plant tolerance to osmotic stress^15^ and to biotic stress^16^. Another pair of stress-inducible *NAC* genes, *SNAC1 & SNAC2* were transcriptionally induced by drought, cold or salt, respectively, and over-expression of these two genes improved rice tolerance to the corresponding abiotic stress(es)^17, 18^. Seven membrane-bound *ZmNTL* genes were identified in maize (*Zea mays*) that were up-regulated upon hydrogen peroxide or abscisic acid treatment suggesting their involvement in abiotic stress tolerance^19^. These previous results from model plants together with featured functional domains (or subdomains) among the *NAC* family genes provide valuable insights into translational research on target gene identification and functional prediction in bioenergy as well as other agronomic crops.

Switchgrass (*Panicum virgatum*), a perennial C_4_ tall grass, is considered as an ideal biomass energy resource due to its high and sustainable biomass production^20, 21^. In order not to compete with grain crops, switchgrass is and will be primarily planted on marginal lands that were often threatened by serious abiotic stresses that will inevitably impose heavy influence on the growth and biomass yield of switchgrass. For examples, switchgrass yield and plant height are severely affected by drought and salt stresses^22^. Transcription factors are key signaling components in the regulation of plant endogenous defense system against abiotic stresses^23, 24^. Therefore, identification of key TFs that enable switchgrass to overcome severe abiotic stresses is an important topic of molecular genetic studies on switchgrass. Yet, due to the self-incompatibility and complex allotetraploid background, it is difficult to pinpoint these key genes in switchgrass using forward genetic approaches. Translational genomics provide an important alternative route for gene identification in switchgrass^25, 26^.

A previous study reported that switchgrass had 107 potential NACs based on unique transcript data^27^. In this study, the much improved switchgrass genome dataset made it possible to identify a better picture of PvNAC family members. Therefore, we conducted a comprehensive genome-wide identification of NAC domain TFs in switchgrass and exploited expanded NAC family with totally 251 family genes. The basic characteristics including sequence phylogeny, gene structure, genome organization, membrane-bound TFs (MTFs), and conserved motif analysis for NAC family of switchgrass were analyzed. We also took advantage of publicly available transcriptomic datasets to systematically analyze *NAC* gene family to identify abiotic stress-related and tissue-specific candidate genes. This study would provide an insight of a large number of candidate *NAC* genes for future genetic studies on switchgrass.

## Results

### Identification and nomenclature of switchgrass NACs

The recently released genome database of “*Panicum virgatum* v1.1, DOE-JGI” and the Hidden Markov Model (HMM) file PF02365 for the NAC domain were used for the identification of PvNACs in this study. A total of 251 switchgrass NAC proteins were identified and designated as PvNAC1 to PvNAC251 (Additional file 1) according to their orders in the chromosomes (The PvNACs started naming from chromosome 1a to 09b), and the rest PvNACs not designated onto its chromosomes were named according to the order of their IDs from the smallest to the largest. The inferred full length of PvNAC proteins ranged from 143 aa to 759 aa, among which 24 proteins were more than 500 aa in length, and 166 proteins were under 300 aa, with molecular weights ranging from 15701.89 to 84903.08 Da, and the isoelectric points from 4.48 to 11.14 (Additional file 1).

### Phylogenetic and structural analyses

A neighbor-joining (N-J) phylogenetic tree was built to show the evolutionary relationship between PvNACs. The PvNAC proteins can be further classified into 19 distinct subgroups (see Supplementary Fig. S1a). The subgroup XIX had the maximum number of PvNACs (29), followed by subgroup IX (23) and XVI (22), while subgroup VII had the least number of PvNACs (4). The structural diversity of *PvNAC* genes was also illustrated with the exon/intron organization (see Supplementary Fig. S1b). And most closely related members in the same subgroups shared highly similar exon/intron structures with comparable intron numbers and exon lengths (see Supplementary Fig. S1b), supporting the subgroup classification of the phylogenetic tree. In addition, a more stringent maximum likelihood approach was also conducted with these 251 PvNACs to confirm the reliability of N-J phylogenetic tree (see Supplementary Fig. S2), and similar results were obtained using both approaches.

**Figure 1:**
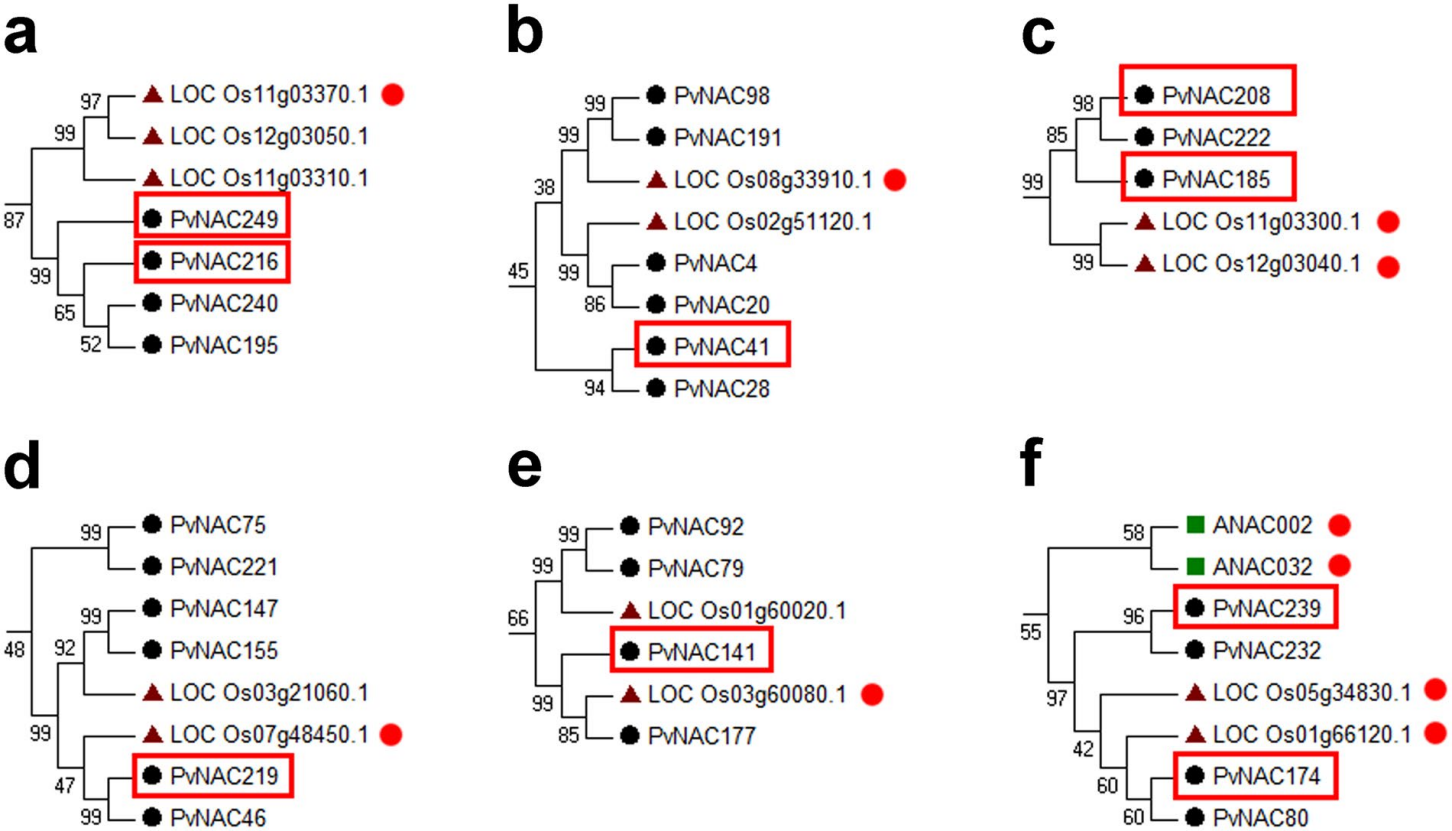
Phylogenetic relationships show the PvNACs that are orthologous to annotated AB- or stress- responsive NAC genes in rice and *Arabidopsis*. (**a**) PvNAC249 and PvNAC216 were orthologous to Os11g03310.1. (**b**) PvNAC41 was orthologous to Os08g339910.1. (**c**) PvNAC208 and PvNAC185 were orthologous to Os11g03300.1 and Os12g03040.1. (**d**) PvNAC219 was orthologous to Os07g48450.1. (**e**) PvNAC141 was orthologous to Os03g60080.1. (**f**) PvNAC239 and PvNAC174 were orthologous to ANAC002, ANAC032, Os05g34830.1, and Os01g66120.1.

Another phylogenetic tree was built from alignments of the full-length PvNACs together with NACs in *Arabidopsis* and rice (see Supplementary Fig. S1c). Accordingly to Ooka *et al*.^8^, most *Arabidopsis* and rice NACs were classified into 18 subgroups, yet a high percentage of switchgrass PvNACs (100; 39.84%) were outside of these subgroups, reflecting the divergence and expansion of specific groups of NACs in switchgrass. For example, eight PvNACs (PvNAC86, −74, −31, −39, −230, −87, and −135) were not grouped with any *Arabidopsis* or rice NAC protein (see Supplementary Fig. S1c), indicating their potential roles in shaping the growth and development or environmental adaptation of switchgrass. Nevertheless, according to the constructed phylogenetic tree and literature review, we were able to pinpoint functional-annotated NACs with their PvNAC homologs (Supplementary Table S1) that are defined as orthologous genes shared similar sequences in a monophyletic group. Accordingly, the potential functions of 27 PvNACs were predicted. These PvNACs can be further clustered into six groups (a–f) (Fig. 1) that were predicted to involve in the regulation of senescence and abiotic stress tolerance.

**Figure 2:**
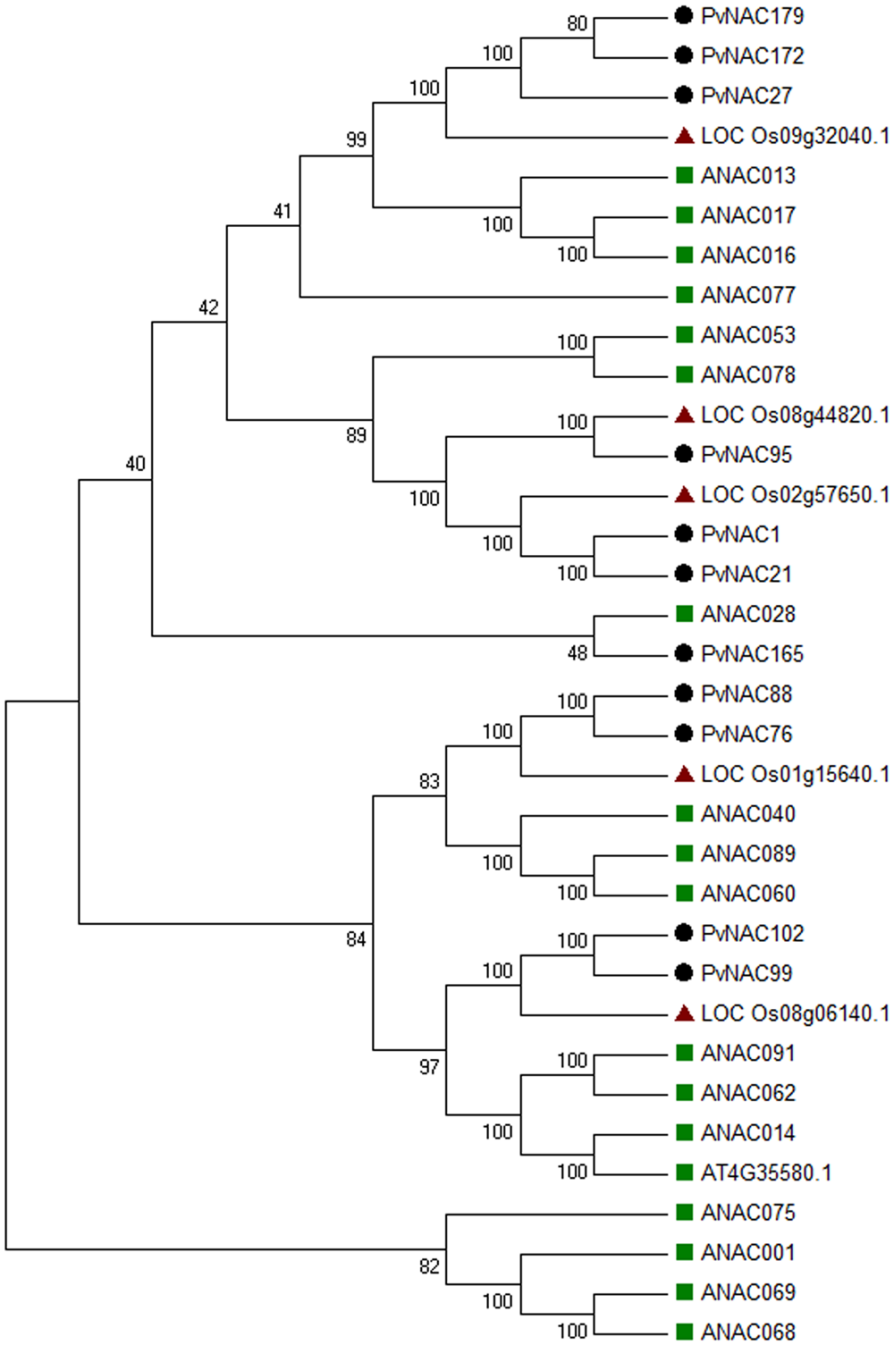
Phylogenetic relationship of NAC MTFs from switchgrass, rice, and *Arabidopsis*. Multiple sequences alignment of NAC MTFs was conducted using ClustalX, and MEGA5.0 was used to construct phylogenetic tree by using Neighbor-joining method with 1000 bootstrap replicates and p-distance method, and bootstrap values are shown next to the branch.

In our study, a total of 11 PvNAC membrane-bound transcription factors (MTFs) were identified by using the TMHMM server 2.0 (Table 1). Each MTF includes an α-helical TM in the C terminal that has a function to anchor onto either endoplasmic reticulum or plasma membranes. Furthermore, four PvNAC MTFs (PvNAC88, −76, −102, and −99) putatively involved in plant stress responses were identified as orthologous ones to the stress-related NAC MTFs of *Arabidopsis* (Fig. 2). While it would be interesting to test whether these four PvNAC MTFs have conserved functions in plant stress tolerance, it was also notable that there was less number of NACs in this clade in grass (switchgrass and rice) than that in *Arabidopsis*.

**Table 1:** Putative membrane-bound switchgrass PvNACs.

Names	Gene model	Length (a.a)	Transmembrane sequences
PvNAC95	Pavir.Fa00048.1	660	629–651
PvNAC165	Pavir.J00978.1	236	188–210
PvNAC99	Pavir.Fa01901.1	674	651–673
PvNAC102	Pavir.Fb00405.1	682	659–681
PvNAC1	Pavir.Aa00085.1	643	619–641
PvNAC21	Pavir.Ab03009.1	636	612–634
PvNAC88	Pavir.Eb01057.1	527	501–520
PvNAC27	Pavir.Ba01681.1	713	681–703
PvNAC179	Pavir.J04800.1	715	683–705
PvNAC76	Pavir.Ea01166.1	531	502–524
PvNAC172	Pavir.J03378.1	632	600–622

### Chromosomal locations and duplications in homeologous chromosomes

**Figure 3:**
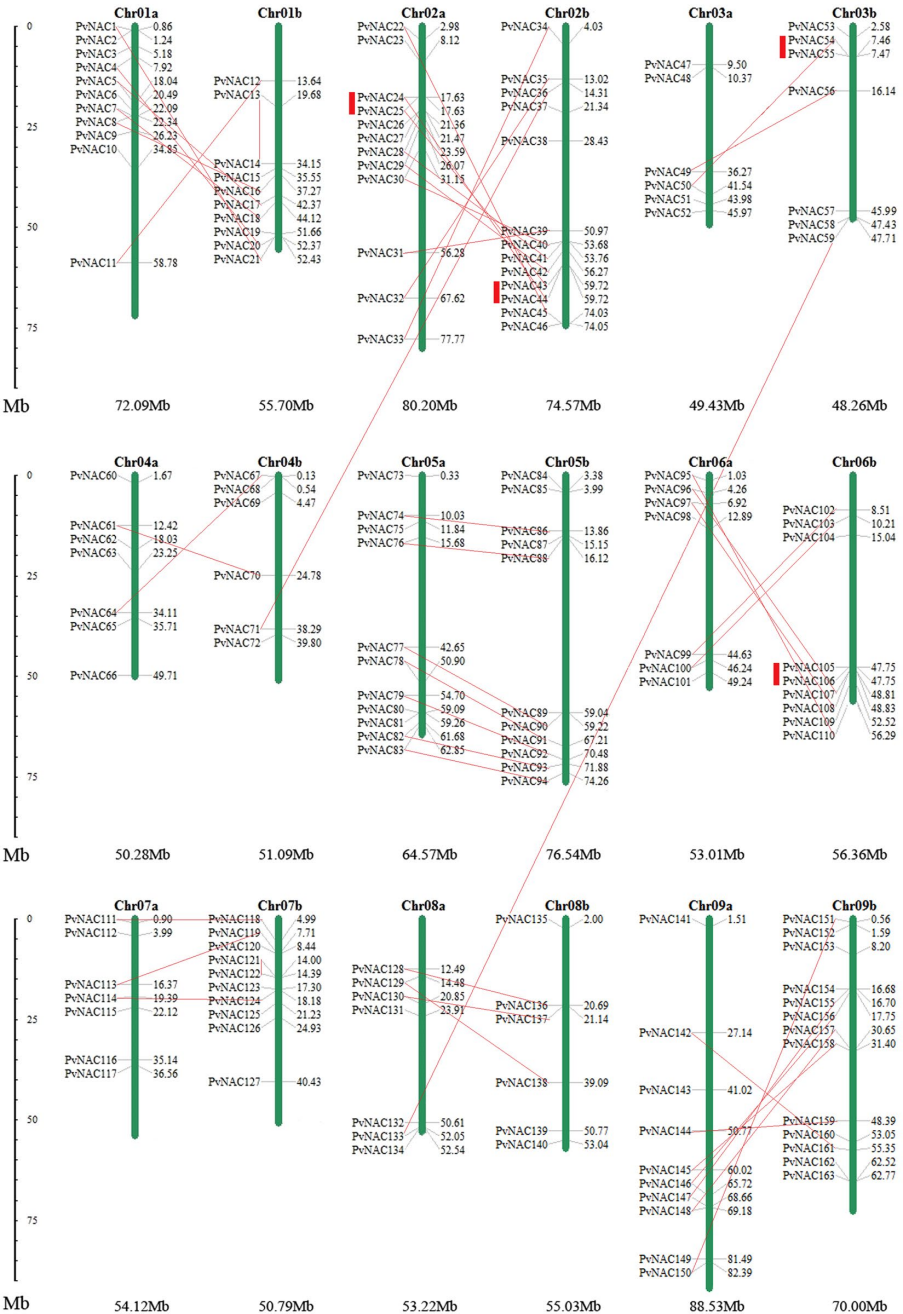
Chromosomal locations of 163 *PvNAC*s. Duplications generated by allotetraploidy were connected by full lines, while tandem duplications were connected by thick red lines. The numbers listed along each chromosome were locations of *PvNAC*s, and the smaller/larger number indicated the *PvNAC*s were closer to start/end point of chromosome. The number below each chromosome was the whole length.

Allotetraploid switchgrass possesses two subgenomes (A, and B)^28^. The segmental duplication and tandem amplification of chromosomal regions are common phenomena that contributing to gene expansion, evolution and diversification of plants^29^. In this study, a total of 163 *PvNAC*s were designated onto 18 chromosomes of switchgrass (Fig. 3), with the rest 88 *PvNAC*s not located on chromosome yet. *PvNAC*s distributed unevenly on chromosomes, with the most on Chr02b and Chr09b (13 *PvNAC*s on each chromosome), and the least on Chr03a, Chr04b, and Chr08b (6 *PvNAC*s). According to the phylogenetic tree, 48 paralogous pairs of *PvNAC* genes were linked with red line (bootstrap value >95 in the phylogenetic tree) as shown in Fig. 3. Majority of these 48 pairs were homeologous chromosomes with only two exceptions (*PvNAC36*/*71*; *PvNAC59*/*133*). Tandem gene duplication was stated that genes in the same chromosome linked in tandem with less than five gene loci. In this study, only four tandem duplications including *PvNAC24*/*PvNAC25* on Chr02a, *PvNAC43*/*PvNAC44* on Chr02b, *PvNAC54*/*PvNAC55* on Chr03b, and *PvNAC105*/*PvNAC106* on Chr06b were discovered (Fig. 3). Together, this result showed that most *PvNAC*s was derived from segmental duplication other than tandem amplification.

Beneficial mutations contribute to species divergence and evolutionary innovation (diversifying selection), and removal of pernicious mutations keeps the natural fitness of species (purifying selection). To understand the evolution trend of these *PvNAC* paralogous genes, we selected 52 pairs of *PvNAC* homologous genes, among which 46 pairs were homeologous, for the calculation of nonsynonymous and synonymous substitution rates (Supplementary Table S2). The result showed that 71.15% (37 out of 52) *PvNAC* pairs were under diversifying selection, and only 15 *PvNAC* pairs (28.85%) under purifying selection.

### Expression profiles of switchgrass NAC genes with public datasets

NACs play important roles in stress tolerance and plant development. Here, we pooled out expression profiles of *PvNAC*s from datasets: a switchgrass Affymetrix array^30^ and a switchgrass gene expression database, PviGEA, and PviUT database^31^.

Using the Affymetrix array data^30^, we re-analyzed the heat-responsive *PvNAC*s. From the total of 199 *PvNAC*s probed on the switchgrass GeneChip (Additional file 2), 24 of them were transcriptionally responsive to heat treatment that three *PvNAC*s were up-regulated, and 21 were down-regulated (see Supplementary Fig. S3).

Tissue-specific gene expression data is useful to identify target genes involved in developmental processes^32^. Taking advantage of the PviGEA and PviUT database^31^, we pooled out and analyzed the expression patterns of 251 *PvNAC*s in 21 differential tissues, organs, and developmental stages (see Supplementary Fig. S4). According to the analysis, genes with specific expression patterns related to lignification, leaf development, flowering, and seed maturation were analyzed.

**Figure 4:**
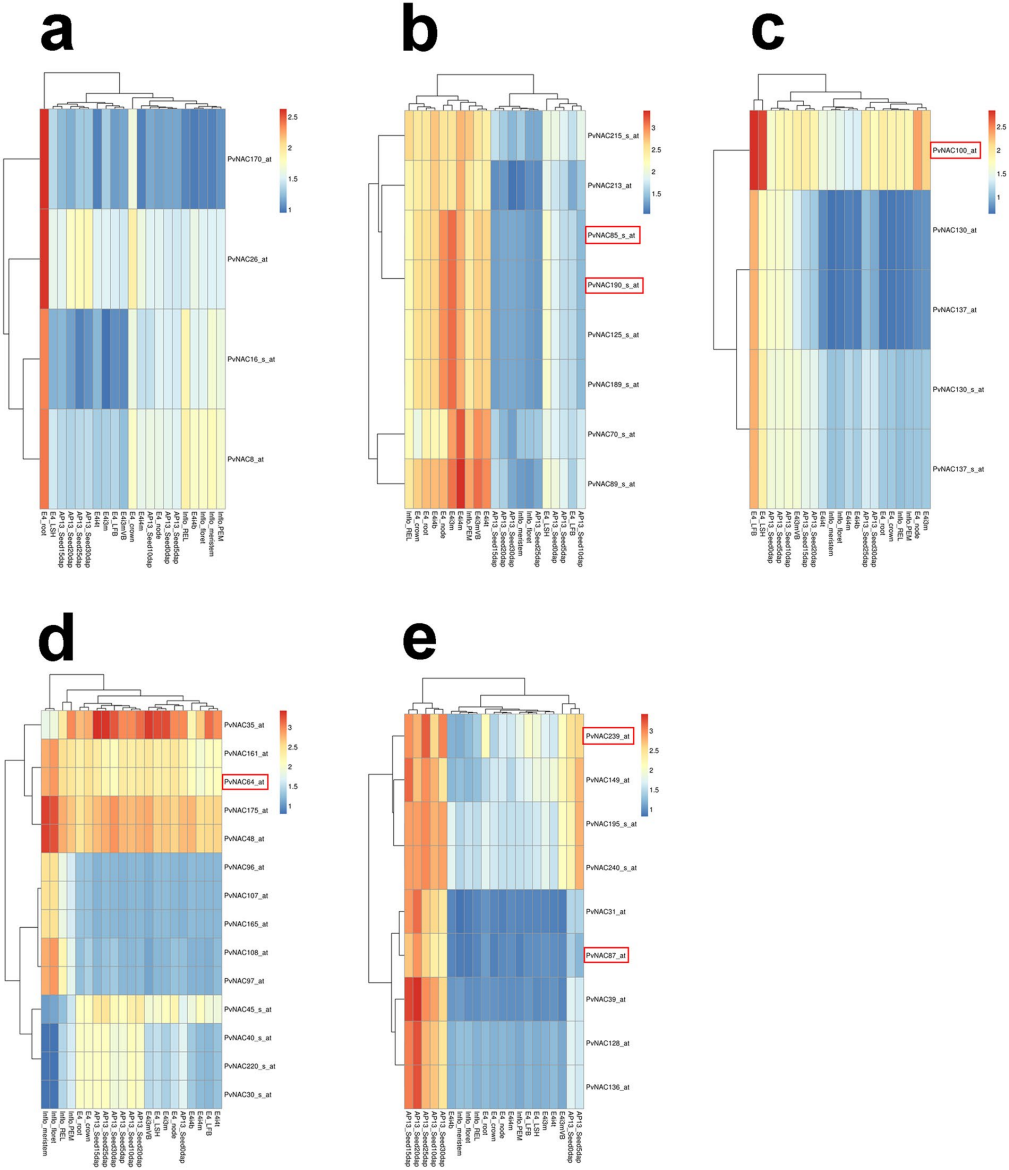
The special expression profiles for several tissues of *PvNAC*s. (**a–e**) Special expression patterns of *PvNAC*s in root, lignified, leave, flower, and seed tissues. AP13_ Seed0d, AP13_ Seed5d, AP13_ Seed10d, AP13_ Seed15d, AP13_ Seed20d, AP13_ Seed25d, AP13_ Seed30d represent whole flowers at anthesis stage, whole seeds 5 days post fertilization, whole seeds with visible caryopsis, whole seeds at the milk stage, whole seeds at the soft dough stage, whole seeds at the hard dough stage, whole seeds at the physiological maturity stage, respectively. Inflo-meristem: Inflorescence meristem (0.5–3.0 mm). Inflo-floret: Floret of inflorescence when glumes are 10–20 mm. Inflo-REL: Rachis and branch elongation of inflorescence (50–150 mm). Inflo-PEM: Panicle emergence of inflorescence (>200 mm). E4-LFB: Pooled leaf blade from plant. E4-LSH: Pooled leaf sheath. E4i3m: Middle 1/5 fragment of the 3rd internode. E4i3mVB: Vascular bundle isolated from 1/5 fragment of the 3rd internode. E4i4b: Bottom 1/5 fragment of the 4th internode. E4i4t: Top 1/5 fragment of the 4th internode. E4i4m: Middle 1/5 fragment of the 4th internode 4. E4-root: Whole root system. E4-crown: Whole crown. E4-node: Pooled nodes. The genes with red frames were chosen for qRT-PCR in further step.

For the *PvNAC*s potentially involved in development of root system, four genes including *PvNAC170*, *−26*, *−16*, and *−8* possess higher expression in root than other tissues (Fig. 4a).

For those potentially involved in the regulation of lignin biosynthesis, we identified eight *PvNAC*s had relatively high expression levels in highly lignified tissues (crown, root, node, internode, and inflorescence branches) (Fig. 4b); and five of these *PvNAC*s (*PvNAC85*, *−89*, *−190*, *−213*, and −*215*) were also previously pinpointed through an orthologous gene identification study^27^. Notably, the rest three *PvNAC*s (*PvNAC70*, *−125*, and *−189*) that showed lignification-related expression patterns were not previously reported and could be novel genes involved in the strengthening of grass cell wall (Supplementary Table S3).

For those potentially involved in cellular metabolism of the green leaves, three *PvNAC*s (*PvNAC100*, *−130*, and *−137*) were identified that had relatively high expression levels in leaves blade and leaf sheath (Fig. 4c).

For those potentially involved in flowering, nine *PvNAC*s were identified (*PvNAC48*, −*64*, −*96*, −*97*, −*107*, −*108*, −*161*, −*165*, and −*175*) that had relative higher expression levels in inflorescence meristem and floret. On the other side, five *PvNAC*s (*PvNAC30*, *−35*, *−40*, *−45*, and *−220*) had obviously lower expression levels in these organs/tissues (Fig. 4d).

*PvNAC*s that might associate with seed development and maturation were also identified: *PvNAC149*, −*195*, −*239* and −*240* had relatively high expression in seed relative to the other tissues. Additionally, *PvNAC31*, −*39*, −*87*, −*128*, and −*136* showed relatively high expression levels from milk stage to physiological maturity stage, but low expression levels from the anthesis stage to five days post fertilization (Fig. 4e), suggesting these genes might involve in the process of seed maturation.

### The interaction network of PvNAC proteins

To further explore the relationship between PvNAC proteins in switchgrass, an interaction network of PvNAC proteins was constructed based on the orthologous rice proteins (see Supplementary Fig. S5; Supplementary Table S4), which identified 15 high confidence interactive proteins involved in the NAC family networks in switchgrass. Through combining with previous analysis as shown in Table S1, seven PvNAC proteins with predicted functions were built in the network (see Supplementary Fig. S5). Specially, PvNAC80, −92, and −141 were all related to drought and/or cell death, indicating that these three NACs coordinately regulate plant drought and/or cell death processes. The pairs between PvNAC147 and −46, and between PvNAC239 and −31 shared similar functions in ABA and drought stresses. PvNAC213 was predicted to interact with PvNAC175 and −89 that these three proteins might regulate secondary cell wall strengthening together. It is notable that this predicted interaction network was based on corresponding rice orthologs. Considering the specificity of different plant species (even though both are in the Poaceae family) and genes’ functional diversification, the reliability of this network shall be further checked through experimental approaches.

### qRT-PCR analysis of selected abiotic stress-responsive and tissue/organ-specific *PvNAC*s

**Figure 5:**
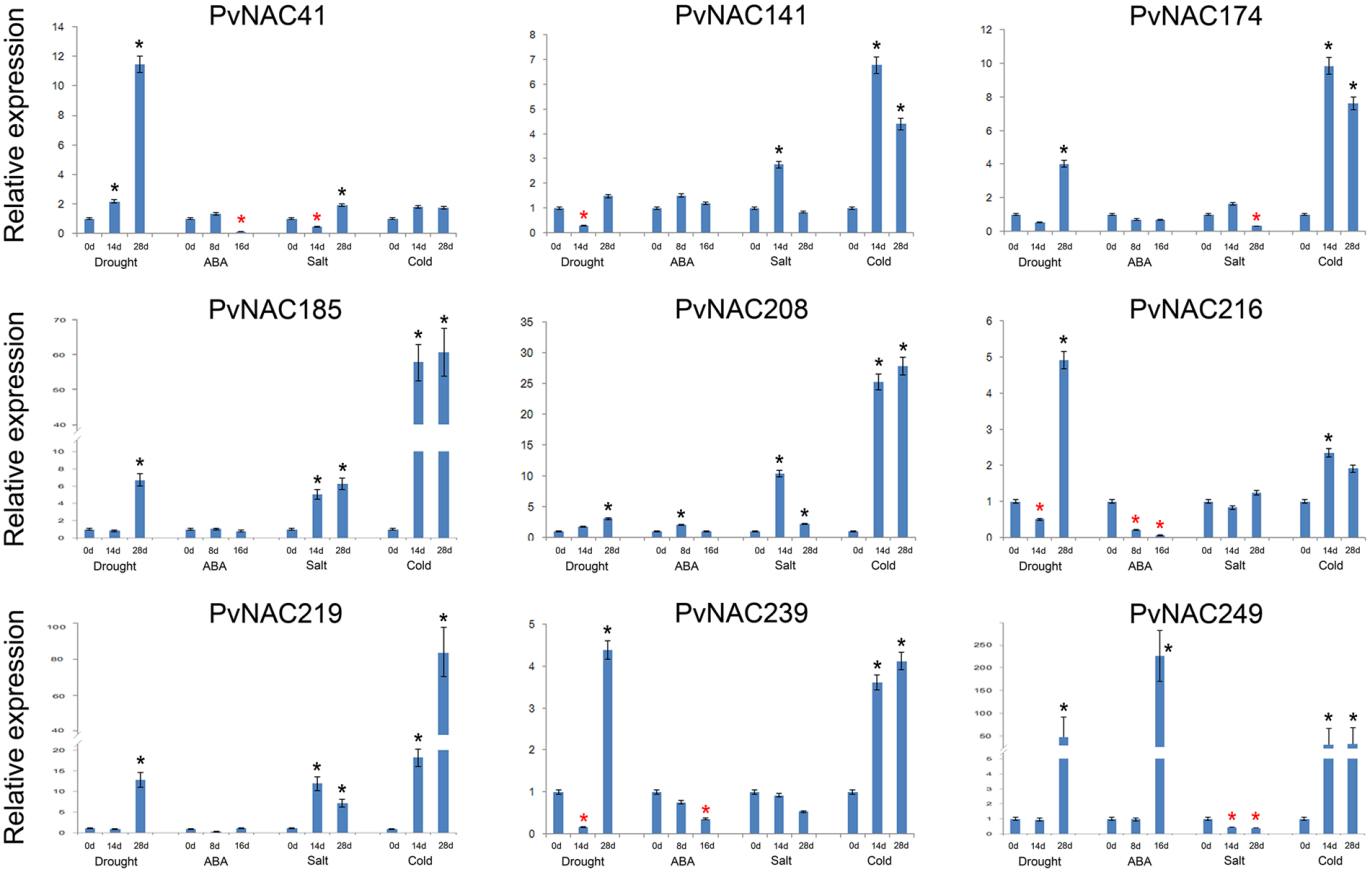
The expression analysis of nine selected *PvNAC*s relating to ABA and drought, salt, and cold stresses via qRT-PCR. Relative expression of these *PvNAC*s were normalized in relation to reference gene *UCE2* in different stresses. The bars represent error bar. The black * represents the expression for treatment group is more than twice as control group (0 d), while red * represents treatment group is below half of the control group (0 d).

The expression patterns of potential stress-related *PvNAC*s were further analyzed using qRT-PCR in response to drought, ABA, salt, and cold treatments (Fig. 5). We selected nine *PvNAC*s (e.g. *PvNAC41*, −*141*, −*174*, −*185*, −*208*, −*216*, −*219*, −*239*, and −*249*) that were orthologous to functional-annotated *NAC* genes involved in plant stress tolerance (Fig. 1) and classified in different subgroups in the phylogenetic tree for gene expression analysis with the gene-specific amplification confirmed by single peak melting curves of the qRT-PCR products (see Supplementary Fig. S6). Setting the cut-off value at 2-fold change, the expression levels of eight *PvNAC*s were significantly up- or down-regulated by three types of treatments, and that of *PvNAC208* were up-regulated by all of the four treatments. Specifically, these nine genes were all significantly up-regulated in switchgrass under severely drought condition (drought for 28 days). The expression of these *PvNAC*s showed different patterns in response to ABA treatment from those to drought treatment that, *PvNAC208* was the only gene transcriptionally up-regulated by ABA treatment after 8 d (2-fold change), while *PvNAC41*, −*216*, and −*239* were significantly down-regulated. When exposed to salt treatment, three genes including *PvNAC185*, −*208*, and *−219* were up-regulated after 14 d and 28 d of treatment, and only *PvNAC249* was significantly down-regulated. Cold treatment significantly induced expression of these genes except *PvNAC41*. In particular, the transcript level of *PvNAC219* and *PvNAC185* increased over 50-fold after 28 d of cold treatment.

**Figure 6:**
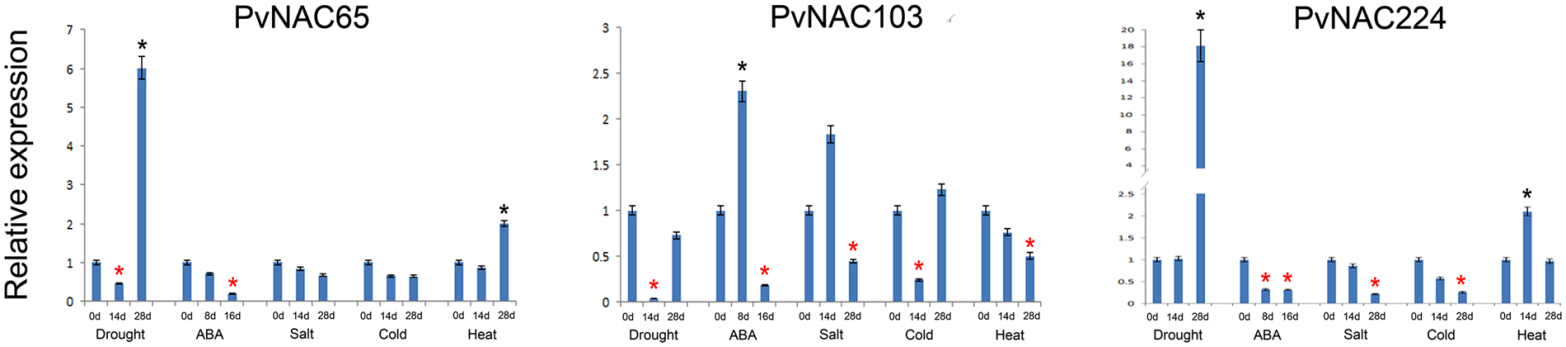
The expression analysis of three selected *PvNAC*s based on array data relating to ABA and drought, salt, cold, and heat stresses through qRT-PCR. Relative expression of these *PvNAC*s were normalized in relation to reference gene *UCE2* in different stresses. The bars represent error bar. The black * represents the expression for treatment group is more than twice as control group (0 d), while red * represents treatment group is below half of the control group (0 d).

**Figure 7:**
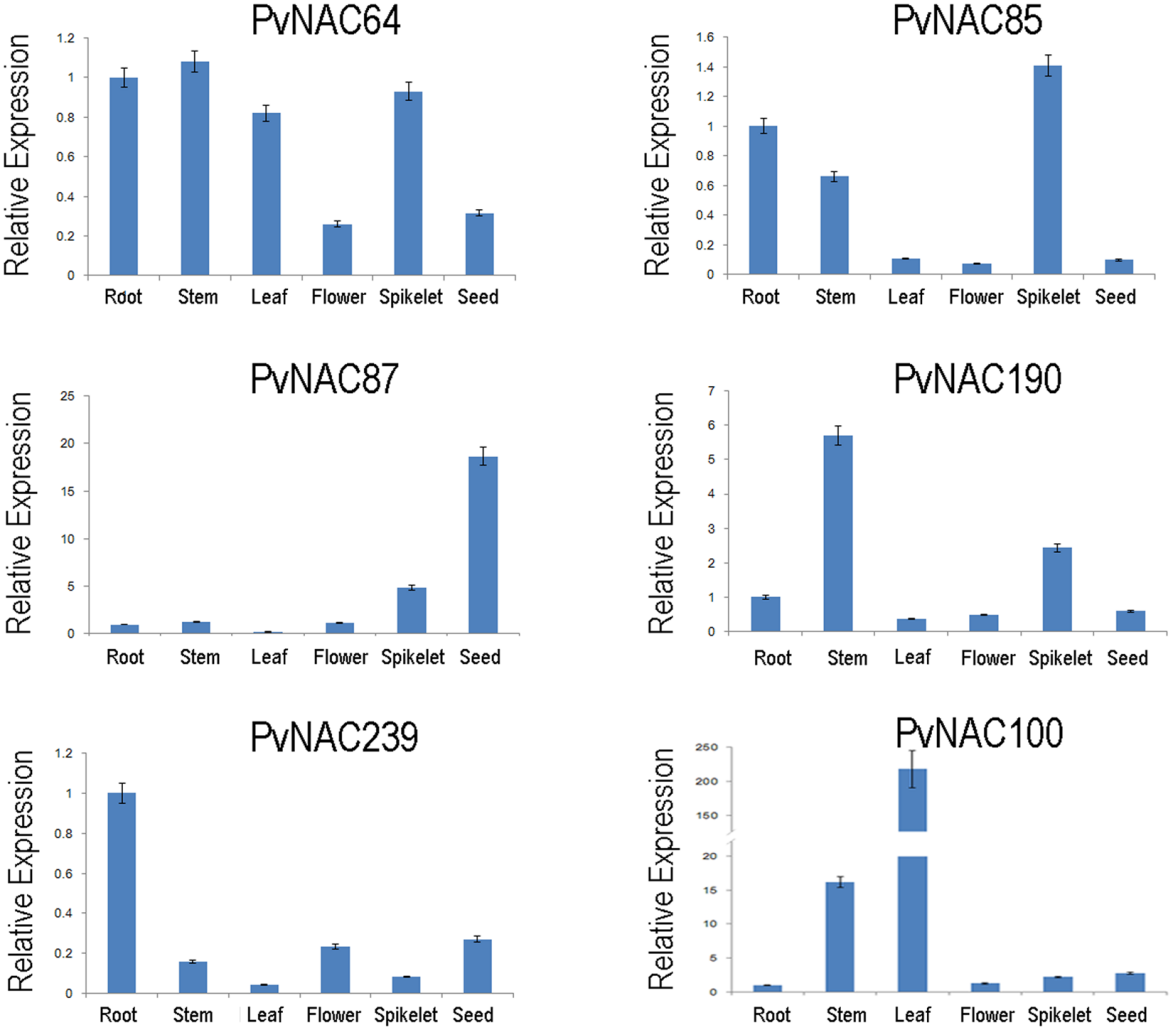
The expression analysis of selected six tissue specific *PvNAC*s via qRT-PCR. Relative expression of these *PvNAC*s were normalized in relation to reference gene *UCE2* in different tissues (Root, stem, leaf, flower, spikelet, seed). The bars represent error bar.

For those 24 *PvNAC*s that showed differential expression upon heat stress as revealed by the Affymetrix data (see Supplementary Fig. S3), we picked three genes (*PvNAC65*, *−103*, and *−224*) to tested their responses to heat, cold, salt, ABA and drought treatments using qRT-PCR (Fig. 6). The results showed that all three genes transcriptionally responded to heat stress at different time points after heat treatment that *PvNAC65* and *−224* transcripts increased to 2-fold after 28 d or 14 d of treatment, yet *PvNAC103* was significantly repressed by heat. Furthermore, these three genes responded to the other abiotic stress treatments as well. For example, the transcript levels of *PvNAC224* and *PvNAC65* increased to 18-fold and 6-fold, respectively, after 28 d of drought treatment. *PvNAC224* was also transcriptionally repressed after prolonged ABA, salt and cold treatments. *PvNAC103* was transcriptionally induced by ABA after 8 d of treatment, but was suppressed by 16 d of treatment, and this gene was also significantly suppressed by drought, salt, and cold after different period of treatment time. To validate the data from the PviGEA and PviUTs database that revealed about 38 *PvNAC*s had tissue/organ-specific express patterns (Fig. 7), we selected six of them with different expression patterns (*PvNAC64*, −*85*, −*87*, −*190*, −*239*, and −*100*) for qRT-PCR analysis. As shown in Fig. 7, the expression levels of tested genes were consistent with the data from PviUT (see Supplementary Fig. S4) in general. For examples, *PvNAC85* and *PvNAC190* had relatively higher expression levels in lignified tissues, such as stem, spikelet and root (Fig. 7). *PvNAC87* had relatively high expression levels in seed, the expression of *PvNAC239* and *PvNAC100* were specifically detected in roots and leaves, respectively. *PvNAC64* displayed significantly lower expression level in flower and seed than the other tissues/organs.

### Correlation between conserved motif analysis and functional predication

NAC proteins share relatively conserved motifs in their N-terminal regions and diversified C-terminals. Therefore, we tried to analyze the conserved motifs among the PvNACs and the correlation between these motifs and their predicted functions.

**Figure 8:**
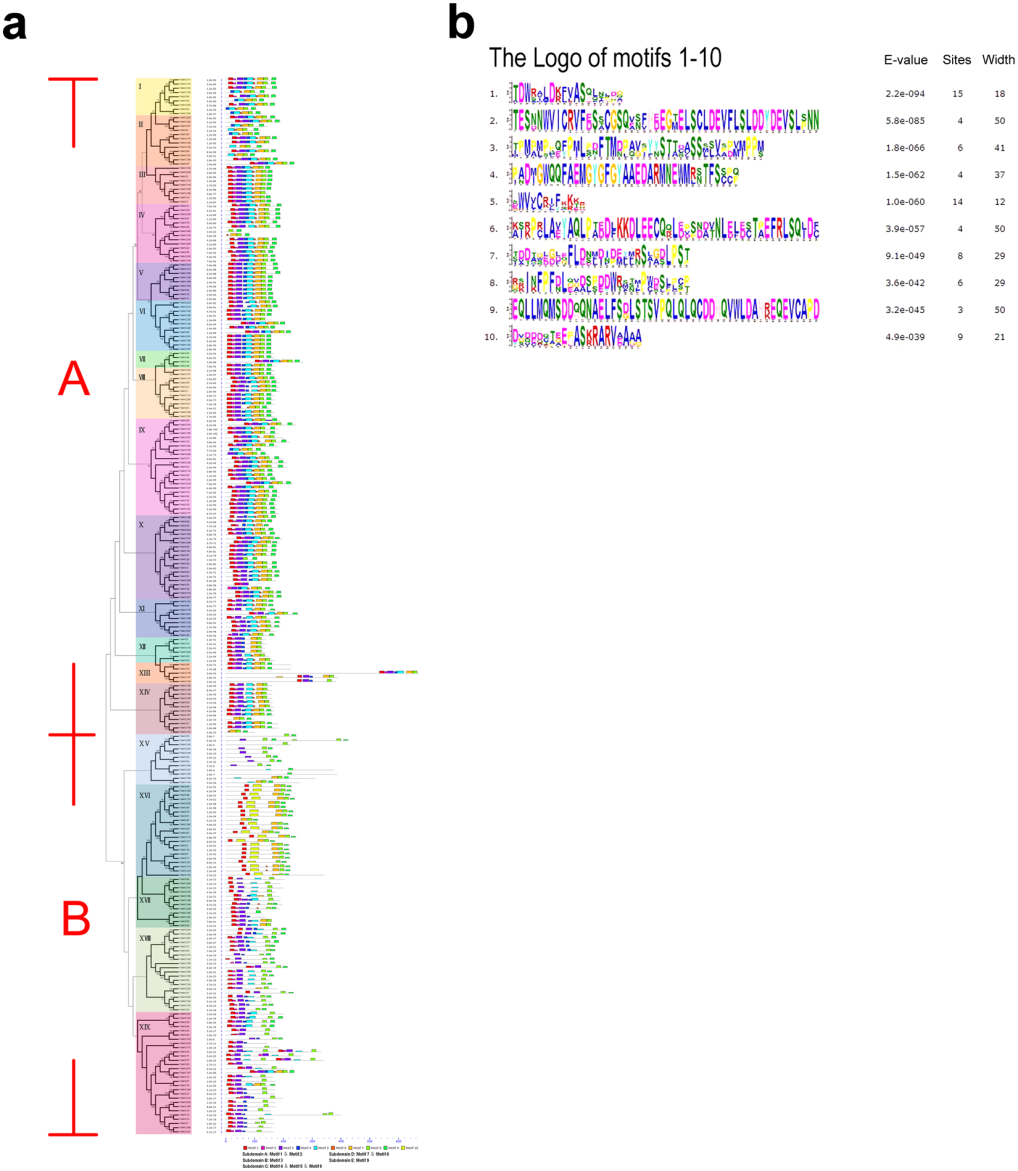
Schematic representation of the conserved motifs in N-terminal regions of PvNACs for displayed by MEME analysis. (**a**) Motifs in N-terminal regions. The colored boxes represent each types of motif, and black lines indicate the non-conserved sequences. (**b**) The logo of motifs 1–10 in N-terminal regions.

As shown in Fig. 8, nearly all PvNACs in Group A had the entire NAC domain (subdomains A-E), while most PvNACs in Group B had incomplete NAC domains that were in lack of one or two subdomains. Among the PvNACs in Group B, only subdomains A was tightly conserved, while subdomains B, C, and E were more divergent, indicating that subdomains A might have the most conserved and indispensable functions for NACs. In particular, PvNACs in the subgroup XV were composed of the least conserved NAC domain that had no subdomain C at all. And it is interesting to note that more than half of PvNACs in subgroup XVI possessed the unique motif 10, and these PvNACs were homologous to ANA010 and ANA073 involved in secondary cell wall thickening in lignified cells^33^ (see Supplementary Fig. S7), indicating that the motif 10 could be an important region in defining the function of these NACs in the process of cell wall biogenesis and lignification.

In the C-terminal of NACs locate TARs (or TRRs) with highly divergent sequences. In this study, a total of 12 motifs (a-l) were identified in the TARs in 10 out of the 13 PvNAC subgroups (see Supplementary Fig. S8), and these motifs in TARS were conserved in parallel with NAC domain structures in the N-terminus. Although the exact roles of these conserved motifs in the TARs of NACs were not well understood, but consistent presence of certain motifs in the C- and N- terminals among certain subgroups in the phylogenetic tree could suggest that these motifs are key components for proper functions of these PvNACs, that hypothesis could be further check using experimental approaches (e.g. domain swap).

## Discussion

### Switchgrass has a large number of unique *NAC* genes under diversifying selection

In this study, we took advantage of the recently released genome database of “*Panicum virgatum* v1.1, DOE-JGI” and identified a total of 251 switchgrass *NAC* genes. The number of *PvNAC*s is the highest among the reported ones, including those in rice (151 *NAC*s)^34^, *Arabidopsis thaliana* (105 *NAC*s)^8^, soybean (*Glycine max*) (152 *NAC*s)^35^, *Populus trichocarpa* (163 *NAC*s)^32^, maize (148 *NAC*s)^36^, and watermelon (*Citrullus lanatus*) (80 *NAC*s)^37^. This large number of *NAC*s in switchgrass could be due to the recent allotetraploidization event at ~1 million years ago (Mya) between two closely related diploid progenitors of switchgrass^38, 39^, and this narrow time frame after the polyploidization event might not be sufficient for large numbers of gene loss and gene diversification and thus provided the genetic basis for the existence of such a large number of *NAC* genes in switchgrass. On the other hand, according to the phylogenetic tree built with NACs of switchgrass, *Arabidopsis* and rice (see Supplementary Fig. S1c), a high percentage of PvNACs (100; 39.84%) were outside of the subgroups assigned for rice and *Arabidopsis* NACs, reflecting the divergence and expansion of specific groups of *NAC* genes in switchgrass. Furthermore, while for 37 pairs of *PvNAC*s under diversifying selection, half of them (51.35%) had dissimilar expression patterns in different organs/developmental stages (see Supplementary Fig. S9).

The tetraploid switchgrass is disomic inheritance with two subgenomes likely originated from a polyploidization event between closely-related diploids^40^. This disomic inheritance displays more opportunities than polysomic inheritance to promote the duplicated genes to undergo divergence and development of new functions^28, 39^. Therefore, the high proportion of *PvNAC* pairs under diversifying selection (71.15%) (Supplementary Table S2) indicated that some *PvNAC*s might have evolved to gain novel or unique functions for the fitness and successful natural adaption of switchgrass.

### Functional predication of PvNACs

Phylogenetic analysis with the whole gene family members is an effective method to predict their potential functions^35, 39^. For example, ZmNAC1 was isolated as the maize otholog to OsNAC6 of rice with 80% of sequence similarity. ZmNAC1 has confirmed to play an important role in stress tolerance against cold, NaCl, drought, and ABA^41^ (Liu *et al*., 2008). Meanwhile, Rabbani *et al*. (2003) suggested that OsNAC6 with a high similarity to ZmNAC1 could also be induced by ABA, cold, salt, and drought stresses^42^. Moreover, NACs were reported to involve in the regulation of biotic and abiotic stress tolerances, plant development, and senescence progress. For examples, OsNAC52 (Os05g34830.1), ONAC045 (Os11g03370.1), SNAC2 (Os01g66120.1), OsNAC10 (Os11g03300.1), ONAC063 (Os08g33910.1) in rice and ATAF1 (ANAC002) in *Arabidopsis* were all positive regulators in plant abiotic stress resistance^18, 43–47^. OsNAC5 (Os11g08210.1) was involved in senescence and its expression was up-regulated with the progression of natural- (aging) and stress-induced senescence (e.g. dark, ABA application, high salinity and cold)^48^. ONAC131 (Os12g03040) was reported to involve in biotic stress response that silencing of ONAC131 yielded rice to be more susceptible to *Magnaporthe grisea* infection (the causal agent of rice blast)^49^. The identification of these conserved switchgrass orthologs to these functional-annotated NACs provide a basis for translating the available knowledge from rice and *Arabidopsis* to switchgrass. In addition, three cell death and stress- related proteins (PvNAC80, −92, and −141) in Table S1 were closely located in protein-protein network (see Supplementary Fig. S5), indicating their mutual effect in functional regulation.

MTFs stored in dormant forms is one regulatory mechanism to quickly respond to environmental stimuli that can be quickly activated upon stimulus signals through degrading the cytoplasmic anchors to enter into the nucleus where they are able to trans-activate or -suppress their target genes^50, 51^. Comprehensive analysis of NAC family predicted 18 and 5 MTFs in *Arabidopsis* and rice, respectively^52^. To date, four *Arabidopsis* NAC MTFs (At3g49530/ANAC062, At2g27300/ANAC040, At4g35580/NTL9 and At4g01540/ANAC068) were activated and liberated from the TM domain by membrane-associated proteases in the endoplasmic reticulum to function in stress responses^51, 53–55^. The PvNAC MTFs possess a single TM that is similar to the NACs in *Arabidopsis*, rice^52^, and it would be interesting to further verify their functions using experimental approaches.

The feedstock quality of switchgrass biomass for bioenergy or forage usage is negatively impacted by the lignin content^56–58^; therefore, identification of genes correlating to the lignin plays an essential part in increasing the conversion efficiency by genetic modification. In this study, we found eight genes with high expression levels in lignified tissues (crown, root, node, internode, and inflorescence branches), but low expression in less lignified tissues (leaf, leaf sheath, florets and seeds) (Fig. 4b), indicating that these eight genes might involve in the process of lignin biosynthesis and/or secondary cell wall strengthening. A previous study also explored switchgrass ten *NAC* genes (corresponding to *PvNAC032*, *−033*, *−046*, *−055*, *−061*, *−062*, *−066*, *−068*, *−101*, and *−102* in this study) that are potential targets for modifying cell wall recalcitrance^27^. In this study, five of them (*PvNAC85*, *−89*, *−190*, *−213*, and *−215*) were also found with higher expression levels in lignified tissues (Fig. 4b), yet the remaining ones did not have that featured expression pattern (*PvNAC16*, *−152*, *−183*).

In order to clearly portrait the *PvNAC* gene expression pattern, the special expression profiles for several tissues were separately analyzed (Fig. 4). The longer and stronger root system plays a significant role in absorbing subsoil surface nutrients and moisture to help plant adapt to drought stress and withhold soil^59^. For *Arabidopsis*, the *AtNAC2* and *NAC1* genes are preferentially overexpressed in roots, and promote lateral root development, which confirm that *AtNAC2* and *NAC1* regulate the lateral root development^60, 61^. Over-expression of the cotton (*Gossypium*) *GhNAC2* under the *CaMV35S* promoter could increase root growth in both *Arabidopsis* and cotton under unstressed conditions^62^. Obviously, in this study, four *PvNAC* genes including *PvNAC170*, *−26*, *−16*, and *−8* possess higher expression in root than the other tissues, indicating that these four genes probably are involved in root development (Fig. 4a).

Leaf photosynthesis, respiration, and senescence are fundamental metabolic processes for plant growth^63–65^. Identification of genes specifically expressed in leaves will be helpful for learning mechanisms in leaf development. For instance, inducible overexpression of *AtNAP*, a gene encoding a NAC family transcription factor in *Arabidopsis*, readily causes precocious leaf senescence^66^. Meanwhile, during drought-induced leaf senescence, a drought-responsive NAC transcription factor NTL4 has been proved to promote ROS production through directly binding to the promoters of genes encoding ROS biosynthetic enzymes in *Arabidopsis* ^67^. Here, leaf special gene expression pattern displayed three genes including *PvNAC100*, *−130*, and *−137* expressed higher in leaf (leaf blade and leaf sheath) than the other tissues (Fig. 4c).

After plants begin to flower, the accumulation of aboveground biomass will decrease^68^. Thus, the delayed flowering time will enable the plants to extend vegetative growth and yield more biomass. In this study, fourteen genes were found to be likely involved in flower development that nine of them had particular higher expression levels in inflorescent floret and five genes had obviously lower expression levels (Fig. 4d).

The low germination rate of switchgrass is another issue hindering its commercialization of seed packages and successful and quick field establishment^69^. Nine genes that may associate with seed development were identified that *PvNAC239*, *−149*, *−195*, and *−240* might involve in seed dormancy or seed maturation at the very late stage, while *PvNAC31*, *−87*, *−39*, *−128*, and *−136* might relate to seed maturation according to their gene expression patterns.

In addition, the current qRT-PCR result supported the previous *in silico* data and provided clues for the functional predication of these *PvNAC*s. Yet, these qRT-PCR analyses had its limitations, e.g. insufficient physiological or phenotype data associated with their relative expression levels and lack of perfect control to normalize the developmental stages across different stress treatments. Further analyses are to be made to confirm functional predications for target genes.

### New insights into motif and structure arrangements

All NAC proteins have relatively conserved NAC domains in the N-terminus with five (A-E) subdomains among which DBDs were contained in subdomains D & E, while subdomains B, C, and E corresponded to the proteins’ diverse functional roles for cooperated DNA-binding specificity of NACs^6, 60, 70^. A previous study showed that subdomains A & E were necessary for stable NAC homo- or hetero-dimer formation, while subdomains D & E indispensable for DNA-binding^71^. The universal presence of subdomain A suggested that the formation of NAC dimers could be essential for the accurate function of NACs. It would be interesting to test the NAC-NAC interaction network to better understand the functional specificity vs functional redundancy among NACs with a special attention to the diversified regions in the future.

In this study, we showed that subdomains B, C, and E were more divergent that might contribute to the functional divergence and specification in certain biological processes. In particular, PvNACs in the subgroup XV were composed of the least conserved NAC domain that had no subdomain C at all. And it is interesting to note that more than half of PvNACs in subgroup XVI possessed the unique motif 10, and these PvNACs were homologous to ANA010 and ANA073 involved in secondary cell wall thickening in lignified cells^33^ (see Supplementary Fig. S6), indicating that the motif 10 could be an important region in defining the function of these NACs in the process of cell wall biogenesis and lignification. Moreover, NACs could also interact with other transcription factors which may be responsible for these differences in the conserved parts of PvNACs (Fig. 8)^72^. The C-terminal of NACs were highly divergent, but short conserved motifs in TARs were also identified that were proposed as core regions for protein interactions and functional specification^8^. In this study, a total of 12 motifs (a-l) were identified in the TARs in 13 out of the 19 PvNAC subgroups (see Supplementary Fig. S8), and these motifs in TARS were conserved in parallel with NAC domain structures in the N-terminus. The exact roles of these conserved motifs in the TARs of NACs were not well understood. If the hypothesis that these motifs function as core regions for protein interactions was valid, then it would be meaningful to further pool out their interacting proteins using these regions as bait by using the Yeast two hybrid system or the other protein interactome technologies.

## Methods

### Database research and sequence retrieval

The switchgrass protein sequences were downloaded from the Phytozome database (http://phytozome.jgi.doe.gov)^73^, and the protein data was built by using HMMER (v 2.3.2). Hidden Markov Model (HMM) profile of NAC domain (PF02365) downloaded from Protein family (Pfam; http://pfam.sanger.ac.uk/)^74^ to be exploited for the identification of the *NAC* genes from local switchgrass database (E-value < 0.001) using HMMER (v 2.3.2). All hits were confirmed by Pfam (PF02365)^74^ and NCBI Conserved Domain Search (http://www.ncbi.nlm.nih.gov/Structure/cdd/wrpsb.cgi)^75^. The confirmed NAC proteins were aligned using Clustal X (v 2.0)^76^ to remove the redundant sequences. Two NAC switchgrass proteins with alternative splicing sites were picked out only with the longest translated protein and the duplicated result was removed in phylogenetic tree analysis. The NAC protein sequences of *Arabidopsis* were downloaded from the *Arabidopsis* genome TAIR 9.0 (http://www.Arabidopsis.org/)^77^ and those of rice retrieved from the Rice Genome Annotation Project website (http://rice.plantbiology.msu.edu/, release 5.0)^78^. Prediction of membrane-bound PvNAC proteins was conducted by using the TMHMM server v.2.0 (http://www.cbs.dtu.dk/services/TMHMM/)^79^.

### Phylogenetic analysis

A total of 251 NAC protein sequences from switchgrass were aligned by Clustal X (v 2.0). MEGA 5.0^80^ was used to construct the unrooted neighbor-joining (N-J) phylogenetic tree (bootstrap 1,000 replicates) based on pairwise gap deletion mode, which was used to certify more different C-terminal domains and could be able to contribute to the topology of the NJ tree. To validate the results from N-J phylogenetic tree, a maximum likelihood method was used to construct the phylogenetic tree based on partial deletion mode. Another phylogenetic tree using the N-J method was built using the same methods for the illustration of relationship between NACs of switchgrass, rice and *Arabidopsis*.

### Genomic structure and motif analysis

The exon-intron display was constructed according to gene structure display server (GSDS) program^81^ according to the available CDS and genomic information of the PvNACs. The conserved motifs among subgroups of PvNACs were identified using the program MEME (Multiple Expectation Maximization for Motif Elicitation; version 4.11.1) (http://meme-suite.org/tools/meme)^82^ with default parameters, and the maximum number of motifs to find was set to 10 for the prediction of A-E subdomains, and 30 for the prediction of TARs^83^.

### Construction of chromosome location images

The chromosomes of switchgrass were ordered to match syntenic fortail millet (*Setaria italica*) chromosome order (http://phytozome.jgi.doe.gov). The chromosome location of PvNACs was generated from MapInspect software according to the available information from the Phytozome database (http://phytozome.jgi.doe.gov). Tandem gene duplication in switchgrass was defined as two paralogs separated by no more than five genes in a range of 100 kb distance on the same chromosome according to the same criteria described in rice (TIGR), and segmental duplications were those placed on replicated chromosomal blocks from the same genome lineage^84^. Moreover, duplications in two sets of homeologous chromosomes can be explained by interspecific genome duplication (allotetraploidy). The selected pairs of homologous genes were used to calculate the ratio between nonsynonymous and synonymous nucleotide substitutions (Ka/Ks) using DNAsp5 software (http://www.ub.edu/dnasp/)^85^.

### Gene expression analysis for transcripts levels in switchgrass tissues and developmental stages

The Unitranscript IDs of the *PvNAC*s were identified in the PviUTs database (http://switchgrassgenomics.noble.org/)^31^. The integrated expression database were obtained by searching against the Switchgrass Gene Expression Atlas (PviGEAs) (http://switchgrassgenomics.noble.org/)^31^. The results were graphically presented in a heatmap format with log fold change after value normalization via the R Project software (http://miyoviqo.tha.im/)^86^. The previously reported cell wall-related NAC genes were used to find homologs among the PvNACs using BLAST.

### Switchgrass affymetrix microarray data analysis under heat stress

For the heat-responsive transcription analysis of the *PvNAC*s, data from the ArrayExpress repository under the accession number E-MTAB-1897^30^ were retrieved. A total of 199 *PvNA*Cs retrieved from the array data were presented in a heatmap with log_2_ fold change after value normalization by the R Project software (http://miyoviqo.tha.im/)^86^.

### Prediction of PvNACs protein-protein interaction network

We constructed an interaction network of PvNAC proteins to explore genome-wide regulation network by using STRING 10 (http://string.embl.de/)^87^ with a default value >0.400, which identified 15 high confidence interactive proteins in rice. The homologs of these interactive proteins in switchgrass were then identified by BLAST analysis.

### Plant material, growth condition and stress treatments

Switchgrass cv. Alamo seeds were sown in pots (0.2 meter diameter × 0.3 m tall) containing 1,000 g soil (pH 5.56, 1.35% organic qualitative content, 100.33 mg/kg N, 4.93 mg/kg P, and 332.25 mg/kg K). The plants were grown in a growth chamber (Wenjiang, Sichuan, China) at 28°/20 °C (day/night) with a photoperiod of 16 h/8 h (day/night). Switchgrass seedlings were thinned to four plants *per* pot after germination. Fifty days after sowing, the potted of switchgrass seedlings were exposed to various stresses including drought, ABA, salt, and cold conditions as follows. For drought treatment, the potted seedlings were maintained without watering for 28 days, and the soil water content of drought-stressed plants was measured to be 10% at the end of drought treatment. And the leaf samples were harvested after 0, 14, 28 days of drought treatment. For ABA treatment, the seedlings were sprayed with 100 mmol ABA for 16 days, and leaves were sampled after 0, 8, 16 days of treatment. For salinity treatment, the seedlings were watered with 250 mmol/l NaCl for 28 days, and leaves were sampled after 0, 14, 28 days of treatment. For cold treatment, the seedlings were subjected to cold stress for 6 °C for 28 days, and leaves were collected after 0, 14, 28 days of treatment. For heat treatment, the plants were exposed to high temperature for 38 °C/30 °C for 28 days and leaves were collected at 0, 14, 28 days. For the tissue/organ-level gene expression profiling, roots, stems, leaves, florets, spikelets, and seeds were collected from field-grown switchgrass, separately. All materials harvested from each treatment were immediately frozen in liquid nitrogen and stored at −80 °C before for RNA isolation. All experiments were conducted three times with three biological replicates for qRT-PCR analysis.

### RNA Isolation, cDNA Synthesis, and Real-time qRT-PCR

Total RNA was extracted using the Total RNA kit II (Qiagen, USA). RNA concentration measurement, DNaseI treatment and cDNA synthesis were conducted as previously described^88^.

Seventeen gene-specific primer pairs (for 12 genes expression analysis under abiotic stresses, and for 6 genes’ in different tissues) were designed by using Primer 5 software^89^ (Supplementary Table S5). We also confirmed the primer specificity by blasting each primer sequence to the switchgrass genome (https://phytozome.jgi.doe.gov/pz/portal.html#!info?alias=Org_Pvirgatum_er; *Panicum virgatum* v1.1). The subsequent analysis of visualization for amplicon fragments and melting curves were performed to confirm whether the 17 primer pairs exhibited an electrophoresis pattern of a single amplicon with accurate length and the corresponding melting curves formed a single sharp peak. In addition, the *Ubiquitin-conjugating enzyme 2* (*UCE2*) gene was selected as reference gene for the expression of switchgrass genes (Supplementary Table S5), and qRT-PCR reactions and data analysis were conducted according to a previous study^88^. The cut-off value of 2-fold for stress-specific expression were adopted in this study. The expression levels were designed as ‘up-regulate’, and ‘down-regulate’ only if the difference conformed to the criteria.

## Conclusion

In conclusion, this study has provided a comprehensive identification and characterization of the switchgrass NAC family. We have identified a total number of 251 NAC proteins in switchgrass genome to be divided into two large groups and 19 subgroups with conserved gene structure. According to the constructed phylogenetic tree with switchgrass, rice, and *Arabidopsis*, a total number of 27 *PvNAC*s were considered to be related to abiotic stresses, and four NAC MTFs were predicted to be orthologous to stress-related MTFs of *Arabidopsis*. In addition, a number of 40 *NAC* genes with tissue-specific expression were found with the help of array data of switchgrass. A number of 163 *PvNAC*s were unevenly distributed on 18 chromosomes, and evolution analysis performed that high proportion of *PvNAC* gene pairs (37/52) were under diversifying selection. The motif analysis showed that all NAC proteins have relatively conserved NAC domains in the N-terminus with five motifs (A-E), while the C-terminal of NACs were highly divergent, but short conserved motifs in TARs were considered as core regions for protein interactions and functional specification. An interaction network of PvNAC proteins was built to predict 15 PvNACs involved in this network, among which seven proteins were potential functional proteins. Next, we designed 17 gene-specific primers for qRT-PCR to confirm 12 *PvNAC*s to be related to various stresses and six *PvNAC*s expressed specifically for different tissues. Even though additional experiments for their under- or over- expression would be helpful for precisely determining the function of these genes, the current results provided useful insights on switchgrass NACs for further genetic engineering studies.

## Electronic supplementary material

Figure S1; Figure S2; Figure S3; Figure S4; Figure S5; Figure S6; Figure S7; Figure S8; Figure S9; Table S1; Table S2; Table S3; Table S4; Table S5

Dataset 1

Dataset 2

## References

[1] Tran LS (2004). Isolation and functional analysis of *Arabidopsis* stress-inducible NAC transcription factors that bind to a drought-responsive cis-element in the early responsive to dehydration stress 1 promoter. Plant Cell..

[2] He XJ (2006). AtNAC2, a transcription factor downstream of ethylene and auxin signaling pathways, is involved in salt stress response and lateral root development. Plant J..

[3] Aida M, Ishida T, Fukaki H, Fujisawa H, Tasaka M (1997). Genes involved in organ separation in Arabidopsis: an analysis of the cup-shaped cotyledon mutant. Plant Cell..

[4] Olsen AN, Ernst HA, Leggio LL, Skriver K (2005). NAC transcription factors: structurally distinct, functionally diverse. Trends Plant Sci..

[5] Puranik S, Sahu PP, Srivastava PS, Prasad M (2012). NAC proteins: regulation and role in stress tolerance. Trends Plant Sci..

[6] Kikuchi K (2000). Molecular analysis of the NAC gene family in rice. Mol Gen Genet..

[7] Jensen MK, Skriver K (2014). NAC transcription factor gene regulatory and protein-protein interaction networks in plant stress responses and senescence. Lubmb Life..

[8] Ooka H (2003). Comprehensive analysis of NAC family genes in *Oryza sativa* and *Arabidopsis thaliana*. Dna Res..

[9] Mitsuda N (2007). NAC transcription factors, NST1 and NST3, are key regulators of the formation of secondary walls in woody tissues of *Arabidopsis*. Plant Cell..

[10] Zhong RQ, Demura T, Ye ZH (2006). SND1, a NAC domain transcription factor, is a key regulator of secondary wall synthesis in fibers of *Arabidopsis*. Plant Cell.

[11] Kubo M (2005). Transcription switches for protoxylem and metaxylem vessel formation. Genes Dev..

[12] Zhong RQ (2015). Functional characterization of NAC and MYB transcription factors involved in regulation of biomass production in switchgrass (Panicum virgatum). Plos One..

[13] Hickman R (2013). A local regulatory network around three NAC transcription factors in stress responses and senescence in *Arabidopsis* leaves. Plant J Cell Mol Biol..

[14] Mohammed N, Sharoni AM, Shoshi K (2013). Roles of NAC transcription factors in the regulation of biotic and abiotic stress responses in plants. Front Microbiol..

[15] Lu PL (2007). A novel drought-inducible gene, ATAF1, encodes a NAC family protein that negatively regulates the expression of stress-responsive genes in *Arabidopsis*. Plant Mol Biol..

[16] Wang X (2009). The *Arabidopsis* ATAF1, a NAC transcription factor, is a negative regulator of defense responses against necrotrophic fungal and bacterial pathogens. Mol Plant Microbe Interact..

[17] Hu HH (2006). Overexpressing a NAM, ATAF, and CUC (NAC) transcription factor enhances drought resistance and salt tolerance in rice. Proc Natl Acad Sci..

[18] Hu HH (2008). Characterization of transcription factor gene SNAC2 conferring cold and salt tolerance in rice. Plant Mol Biol..

[19] Wang D (2016). Membrane-bound NAC transcription factors in maize and their contribution to the oxidative stress response. Plant Sci..

[20] Parrish DJ, Fike JH (2005). The biology and agronomy of switchgrass for biofuels. CRC Crit Rev Plant Sci..

[21] Shield I (2008). Bioenergy from plants and the sustainable yield challenge. New Phytol..

[22] Fike JH (2006). Long-term yield potential of switchgrass-for-biofuel systems. Biomass Bioenergy.

[23] Singh KB, Foley RC, Oñate-Sánchez L (2002). Transcription factors in plant defense and stress responses. Curr Opin Plant Biol..

[24] Li J (2013). Defense‐related transcription factors WRKY70 and WRKY54 modulate osmotic stress tolerance by regulating stomatal aperture in *Arabidopsis*. New Phytol..

[25] Frazier TP (2016). Identification, characterization, and gene expression analysis of nucleotide binding site (NB)-type resistance gene homologues in switchgrass. BMC Genomics..

[26] Rinerson CI (2015). The WRKY transcription factor family and senescence in switchgrass. BMC Genomics..

[27] Shen H, Yin YB, Chen F, Xu Y, Dixon RA (2009). A Bioinformatic analysis of NAC genes for plant cell wall development in relation to lignocellulosic bioenergy production. Bioenerg Res..

[28] Okada M (2010). Complete switchgrass genetic maps reveal subgenome collinearity, preferential pairing and multilocus interactions. Genet..

[29] Leister D (2004). Tandem and segmental gene duplication and recombination in the evolution of plant disease resistance gene. Trends Genet..

[30] Li YF, Wang YX, Tang YH, Kakani VG, Mahalingam R (2013). Transcriptome analysis of heat stress response in switchgrass (*Panicum virgatum* L.). Bmc Plant Biol..

[31] Zhang JY (2013). Development of an integrated transcript sequence database and a gene expression atlas for gene discovery and analysis in switchgrass (*Panicum virgatum* L.). Plant J..

[32] Hu RB (2010). Comprehensive analysis of NAC domain transcription factor gene family in *Populus trichocarpa*. BMC Plant Biol..

[33] Zhong RQ, Lee CH, Zhou JL, Mccarthy RL, Ye ZH (2008). A battery of transcription factors involved in the regulation of secondary cell wall biosynthesis in *Arabidopsis*. Plant Cell..

[34] Nuruzzaman M (2010). Genome-wide analysis of NAC transcription factor family in rice. Gene..

[35] Le DT (2011). Genome-wide survey and expression analysis of the plant-specific NAC transcription factor family in soybean during development and dehydration stress. Dna Res..

[36] Peng XJ (2015). Genomewide identification, classification and analysis of NAC type gene family in maize. J Genet..

[37] Lv, X. L. *et al*. Global expressions landscape of NAC transcription factor family and their responses to abiotic stresses in *Citrullus lanatus*. *Sci Rep*. **6**, doi:10.1038/srep30574 (2016). 10.1038/srep30574 PMC4974498 27491393

[38] Huang SX, Su XJ, Haselkorn R, Gornicki P (2003). Evolution of switchgrass (*Panicum virgatum* L.) based on sequences of the nuclear gene encoding plastid acetyl-CoA carboxylase. Plant Sci..

[39] Yuan SX (2015). Comprehensive analysis of CCCH-type zinc finger family genes facilitates functional gene discovery and reflects recent allopolyploidization event in tetraploid switchgrass. BMC Genomics..

[40] Lu F (2013). Switchgrass genomic diversity, ploidy, and evolution: novel insights from a network-based SNP discovery protocol. PLoS Genet..

[41] Liu ZJ, Shao FX, Tang GY, Shan L, Bi YP (2009). Cloning and characterization of a transcription factor ZmNAC1 in maize (*Zea mays*). Hereditas..

[42] MA R (2003). Monitoring expression profiles of rice genes under cold, drought, and high-salinity stresses and abscisic acid application using cDNA microarray and RNA gel-blot analyses. Plant Physiol..

[43] Gao F (2010). OsNAC52, a rice NAC transcription factor, potentially responds to ABA and confers drought tolerance in transgenic plants. Plant Cell Tissue Organ Cult.

[44] Zheng XN, Bo C, Lu GJ, Han B (2009). Overexpression of a NAC transcription factor enhances rice drought and salt tolerance. Biochem Biophys Res Commun.

[45] Jin Seo J (2010). Root-specific expression of OsNAC10 improves drought tolerance and grain yield in rice under field drought conditions. Plant Physiol..

[46] Yokotani N (2009). Tolerance to various environmental stresses conferred by the salt-responsive rice gene ONAC063 in transgenic. Arabidopsis. Planta.

[47] Wu YR (2009). Dual function of *Arabidopsis* ATAF1 in abiotic and biotic stress responses. Cell Res..

[48] Sperotto RA (2009). Identification of up-regulated genes in flag leaves during rice grain filling and characterization of Os NAC5, a new ABA-dependent transcription factor. Planta..

[49] Sun LJ (2013). Functions of rice NAC transcriptional factors, ONAC122 and ONAC131, in defense responses against *Magnaporthe grisea*. Plant Mol Biol.

[50] Hoppe T (2000). Activation of a membrane-bound transcription factor by regulated ubiquitin/proteasome-dependent processing. Cell..

[51] Kim SY (2007). Exploring membrane-associated NAC transcription factors in *Arabidopsis*: implications for membrane biology in genome regulation. Nucleic Acids Res..

[52] Kim SG (2010). Genome-scale screening and molecular characterization of membrane-bound transcription factors in *Arabidopsis* and rice. Genom..

[53] Seo PJ, Kim SG, Park CM (2008). Membrane-bound transcription factors in plants. Trends Plant Sci..

[54] Kim SG, Lee AK, Yoon HK, Park CM (2008). A membrane-bound NAC transcription factor NTL8 regulates gibberellic acid-mediated salt signaling in *Arabidopsis* seed germination. Plant J..

[55] Chen YN, Slabaugh E, Brandizzi F (2008). Membrane-tethered transcription factors in *Arabidopsis thaliana*: novel regulators in stress response and development. Curr Opin Plant Biol..

[56] Fu C (2011). Genetic manipulation of lignin reduces recalcitrance and improves ethanol production from switchgrass. Proc Natl Acad Sci..

[57] Samuel R, Foston M, Jiang N, Allison L, Ragauskas AJ (2011). Structural changes in switchgrass lignin and hemicelluloses during pretreatments by NMR analysis. Polym Degrad Stab..

[58] Chen F, Dixon RA (2007). Lignin modification improves fermentable sugar yields for biofuel production. Nat Biotechnol..

[59] Achard P (2006). Integration of plant responses to environmentally activated phytohormonal signals. Sci..

[60] Xie Q, Frugis G, Colgan D, Chua NH (2000). Arabidopsis NAC1 transduces auxin signal downstream of TIR1 to promote lateral root development. Genes Dev..

[61] He XJ (2005). AtNAC2, a transcription factor downstream of ethylene and auxin signaling pathways, is involved in salt stress response and lateral root development. Plant J..

[62] Gunapati, S. *et al*. Corrigendum: Expression of *GhNAC2* from *G. herbaceum*, improves root growth and imparts tolerance to drought in transgenic cotton and *Arabidopsis*. *Sci Rep*. **6**, doi:10.1038/srep34897 (2016). 10.1038/srep34897 PMC5056452 27721386

[63] Gulmon SL, Chu CC (1981). The effects of light and nitrogen on photosynthesis, leaf characteristics and dry matter allocation in the Chaparral shrub *Diplacus aurantiacus*. Oecologia..

[64] Atkin OK, Evans JR, Ball MC, Lambers H, Pons TL (2000). Leaf respiration of snow gum in the light and dark. Interactions between temperature and irradiance. Plant Physiol..

[65] Estiarte M, Peñuelas J (2014). Alteration of the phenology of leaf senescence and fall in winter deciduous species by climate change: effects on nutrient proficiency. Glob Chang Biol..

[66] Guo YF, Gan SS (2006). AtNAP, a NAC family transcription factor, has an important role in leaf senescence. Plant J..

[67] Lee S, Seo PJ, Lee HJ, Park CM (2012). A NAC transcription factor NTL4 promotes reactive oxygen species production during drought-induced leaf senescence in *Arabidopsis*. Plant J..

[68] Van Esbroeck GA (1997). Leaf appearance rate and final leaf number of switchgrass cultivars. Crop Sci..

[69] Aiken GE, Springer TL (1995). Seed size distribution, germination, and emergence of 6 switchgrass cultivars. J Range Manag..

[70] Wang BY (2015). Identification and characterization of plant-specific NAC gene family in canola (*Brassica napus* L.) reveal novel members involved in cell death. Plant Mol Biol..

[71] Taoka K-i (2004). The NAC domain mediates functional specificity of CUP-SHAPED COTYLEDON proteins. Plant J.

[72] Olsen AN, Ernst HA, Leggio LL, Skriver K (2005). NAC transcription factors: structurally distinct, functionally diverse. Trends in Plant Science..

[73] Goodstein DM (2011). Phytozome: a comparative platform for green plant genomics. Nucleic Acid Res..

[74] Finn RD (2014). Pfam: the protein families database. Nucleic Acids Res.

[75] Marchler-Bauer A (2011). CDD: a Conserved Domain Database for the functional annotation of proteins. Nucleic Acid Res..

[76] Larkin MA (2007). Clustal W and Clustal X version 2.0. Bioinform..

[77] Rhee SY (2003). The *Arabidopsis* information resource (TAIR): a model organism database providing a centralized, curated gateway to Arabidopsis biology, research materials and community. Nucleic Acid Res.

[78] Kawahara Y (2013). Improvement of the *Oryza sativa* Nipponbare reference genome using next generation sequence and optical map data. Rice..

[79] Gu L, Rose K (2000). Sub-state tying in tied mixture hidden Markov models. Lcassp..

[80] Tamura K (2011). MEGA5: molecular evolutionary genetics analysis using maximum likelihood, evolutionary distance, and maximum parsimony methods. Mol Biol Evol..

[81] Guo AY, Zhu QH, Chen X (2007). GSDS:a gene structure display server. Yi Chuan..

[82] Bailey TL (2009). MEME SUITE: tools for motif discovery and searching. Nucleic Acid Res..

[83] Bailey TL, Williams N, Misleh C, Li WW (2006). MEME: discovering and analyzing DNA and protein sequence motifs. Nucleic Acids Res..

[84] Peng XJ (2012). CCCH-type zinc finger family in maize: genome-wide identification, classification and expression profiling under abscisic acid and drought treatments. Plos One..

[85] Librado P, Rozas J (2009). DnaSP v5: a software for comprehensive analysis of DNA polymorphism data. Bioinform.

[86] Ripley BD (2001). The R project in statistical computing. Msor Connect..

[87] Szklarczyk D (2015). STRING v10: protein–protein interaction networks, integrated over the tree of life. Nucleic Acid Res..

[88] Huang LK (2014). Evaluation of candidate reference genes for normalization of quantitative RT-PCR in switchgrass under various abiotic stress conditions. Bioenergy Res..

[89] Lalitha S (2000). Primer Premier 5. Biotech Softw Internet Rep..

