# Supplementary material for: Genome-wide survey of switchgrass NACs family provides new insights into motif and structure arrangements and reveals stress-related and tissue-specific NACs
Source: Sci Rep. 2017 Jun 8;7:3056. doi: 10.1038/s41598-017-03435-z (PMC5465074; doi:10.1038/s41598-017-03435-z)
Supplement: Supplementary file 1 — Figure S1; Figure S2; Figure S3; Figure S4; Figure S5; Figure S6; Figure S7; Figure S8; Figure S9; Table S1; Table S2; Table S3; Table S4; Table S5 [file 41598_2017_3435_MOESM1_ESM.pdf]

Genome-wide survey of switchgrass NACs family provides new insights into motif and structure arrangements and reveals stress-related and tissue-specific NACs

Haidong Yan[1], Ailing Zhang[1], Yuntian Ye[2], Bin Xu[3,*], Jing Chen[1], Xiaoyan He[1], Chengran Wang[1], Sifan Zhou[1], Xinquan Zhang[1,*], Yan Peng[1], Xiao Ma[1], Yanhong Yan[1], Linkai Huang[1,*].

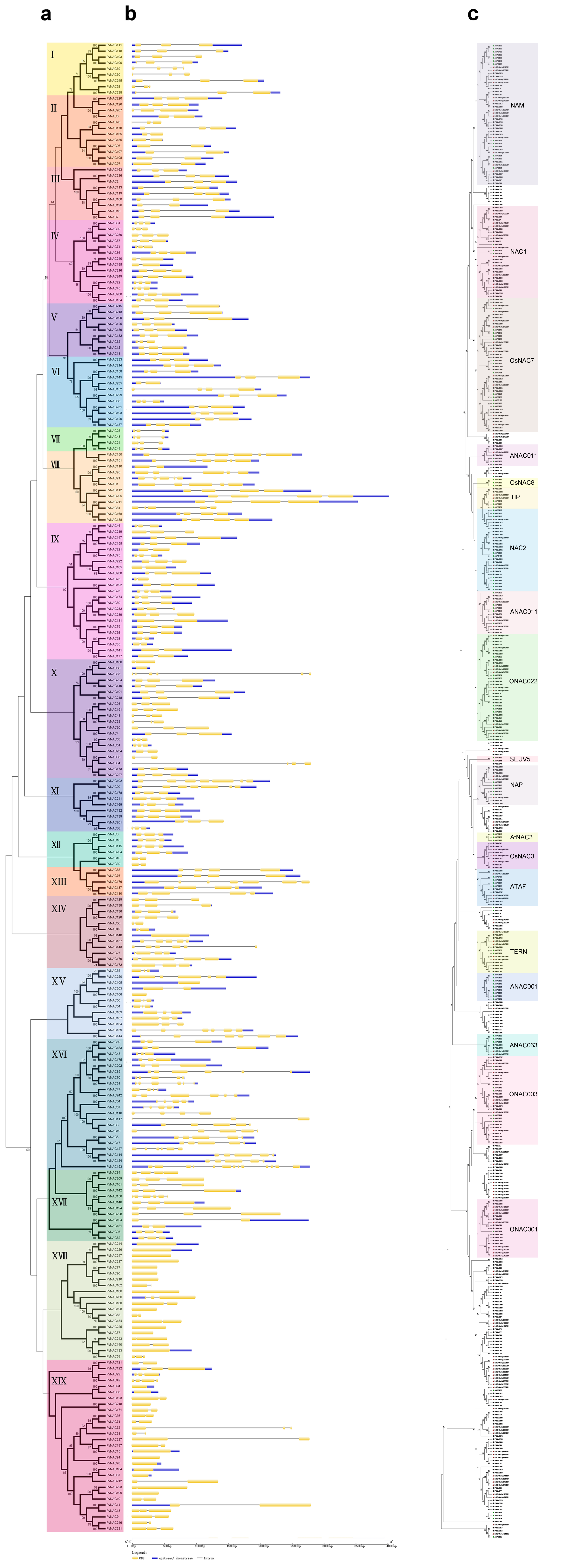

a
b
c
I
II
III
IV
V
VI
VII
VIII
IX
X
XI
XII
XIII
XIV
XV
XVI
XVII
XVIII
XIX
NAM
NAC1
OsNAC7
ANAC011
OsNAC8
TIP
NAC2
ANAC011
ONAC022
SEUV5
NAP
AtNAC3
OsNAC3
ATAF
TERN
ANAC001
ANAC063
ONAC003
ONAC001

Figure S1 Analysis of phylogenetic relationships for NAC genes. a. Multiple sequence alignment of 251 amino acids of PvNACs was conducted using ClustalX, and the phylogenetic tree was constructed via MEGA 5.0 by the Neighbor-joining method with 1000 bootstrap replicates and p-distance method. The phylogenetic tree only showed percentage bootstrap scores above 50%. A number of 19 phylogenetic subgroups were divided as I to XIX marked with different colour backgrounds. b. Exon/intron structures of *PvNAC*s. Exons and introns are yellow boxes and black lines, respectively, and blue boxes indicate upstream/downstream sequence. c. Phylogenetic relationships of switchgrass, rice and *Arabidopsis* NAC proteins. We classified the NACs into 18 subgroups, and name of subgroups are followed by Ooka et al. (2003).

Reference

Ooka, H. et al. Comprehensive analysis of NAC family genes in Oryza sativa and Arabidopsis thaliana. Dna Res. 10, 239-47 (2003).

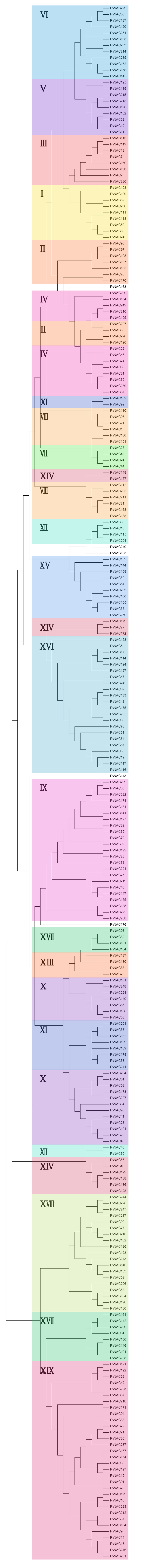
VI
V
III
I
II
IV
II
IV
XI
VIII
VII
XIV
VIII
XII
XV
XIV
XVI
IX
XVII
XIII
X
XI
X
XII
XIV
XVIII
XVII
XIX

Figure S2 Phylogenetic relationships of switchgrass NAC proteins. Multiple sequence alignment of NACs was conducted using ClustalX, and the phylogenetic tree was constructed via MEGA 5.0 by the Maximum likelihood method based on partial deletion mode. The classification of this figure was in accordance with the figure S1a.

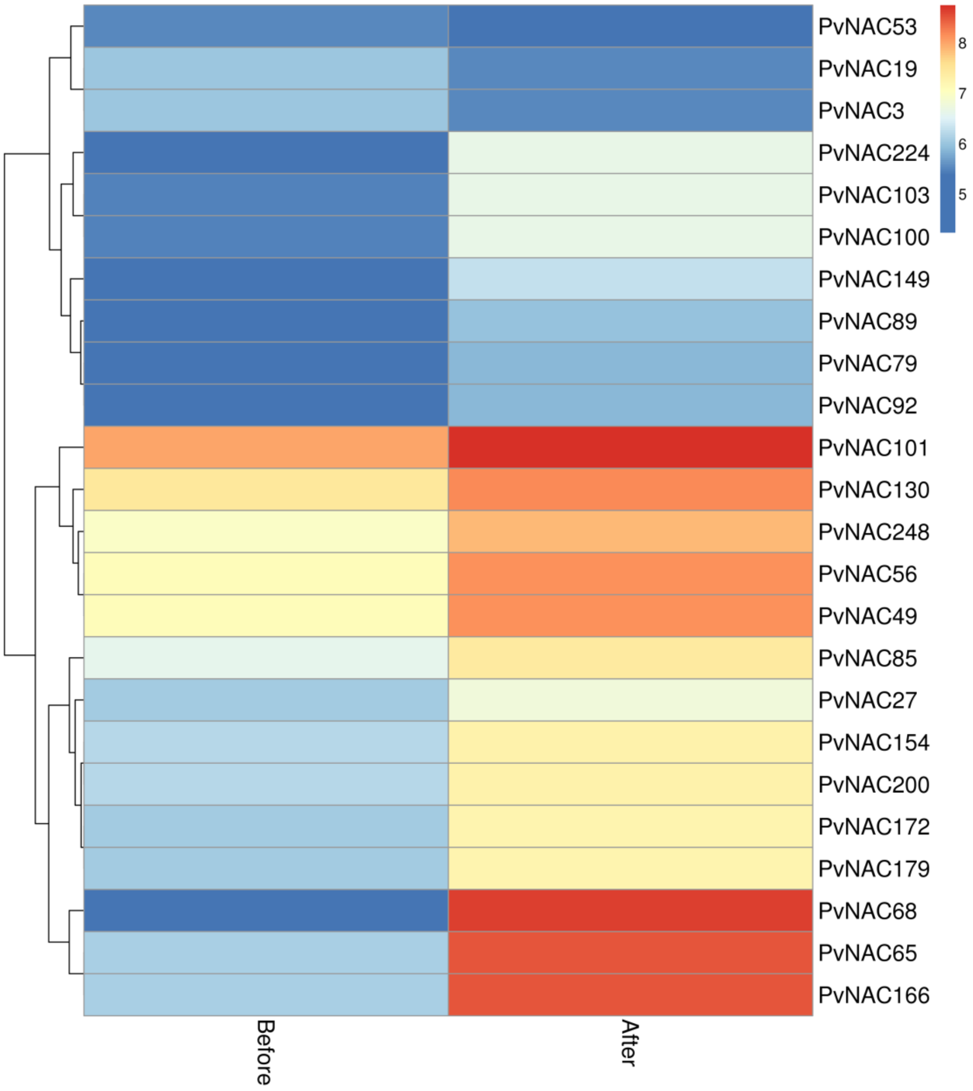

Figure S3 The special expression profiles of 24 *PvNAC*s upon heat stress as revealed by the Affymetrix data.

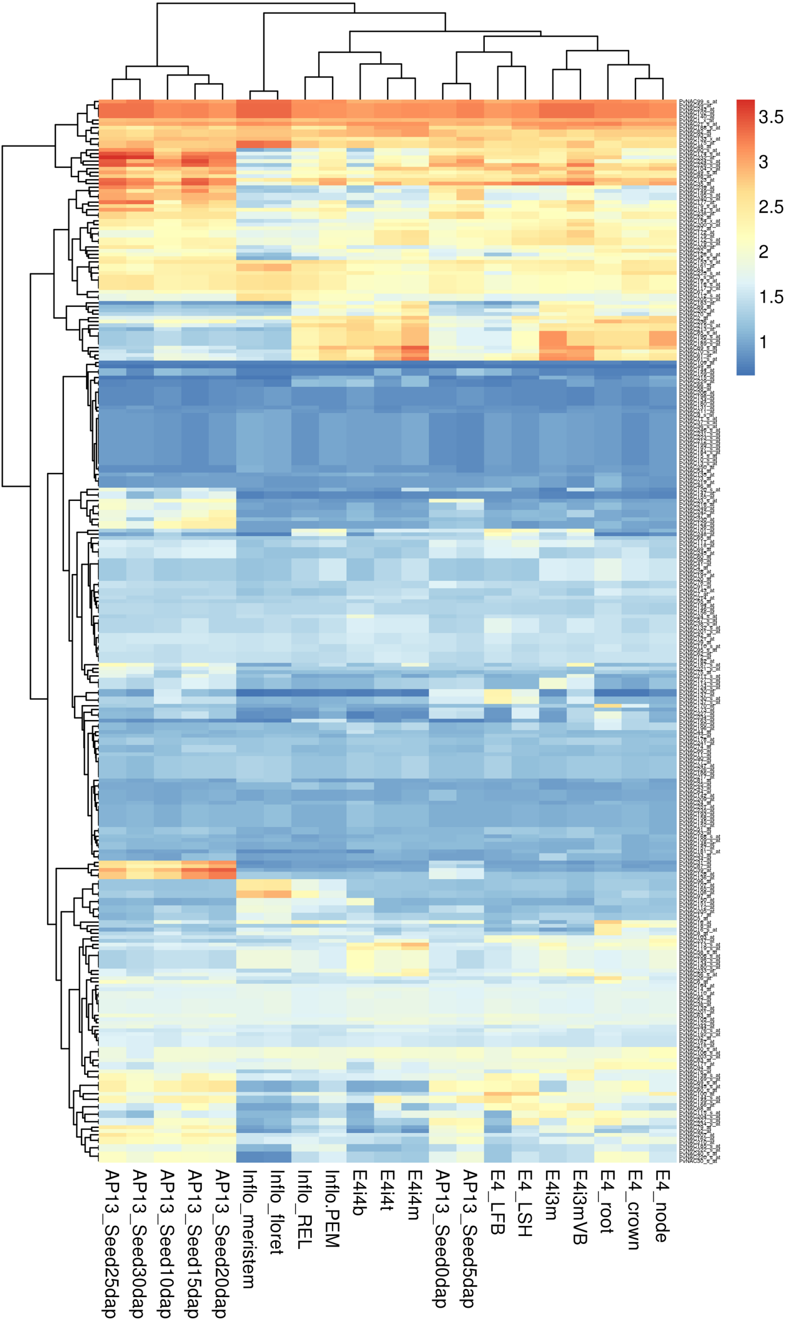
3.5
3
2.5
2
1.5
1
AP13_Seed25dap
AP13_Seed30dap
AP13_Seed10dap
AP13_Seed15dap
AP13_Seed20dap
Inflo_meristem
Inflo_floret
Inflo_REL
Inflo.PEM
E4i4b
E4i4t
E4i4m
AP13_Seed0dap
AP13_Seed5dap
E4_LFB
E4_LSH
E4i3m
E4i3mVB
E4_root
E4_crown
E4_node

Figure S4 Heatmap of expression profiles of *PvNAC*s in 21 organs, tissues or at different developmental stages. AP13_ Seed0d, AP13_ Seed5d, AP13_ Seed10d, AP13_ Seed15d, AP13_ Seed20d, AP13_ Seed25d, AP13_ Seed30d represent whole flowers at anthesis stage, whole seeds 5 days post fertilization, whole seeds with visible caryopsis, whole seeds at the milk stage, whole seeds at the soft dough stage, whole seeds at the hard dough stage, whole seeds at the physiological maturity stage, respectively. Inflo-meristem: Inflorescence meristem (0.5-3.0 mm). Inflo-floret: Floret of inflorescence when glumes are 10–20 mm. Inflo-REL: Rachis and branch elongation of inflorescence (50–150 mm). Inflo-PEM: Panicle emergence of inflorescence (>200 mm). E4-LFB: Pooled leaf blade from plant. E4-LSH: Pooled leaf sheath. E4i3m: Middle 1/5 fragment of the 3rd internode. E4i3mVB: Vascular bundle isolated from 1/5 fragment of the 3rd internode. E4i4b: Bottom 1/5 fragment of the 4th internode. E4i4t: Top 1/5 fragment of the 4th internode. E4i4m: Middle 1/5 fragment of the 4th internode 4. E4-root: Whole root system. E4-crown: Whole crown. E4-node: Pooled nodes.

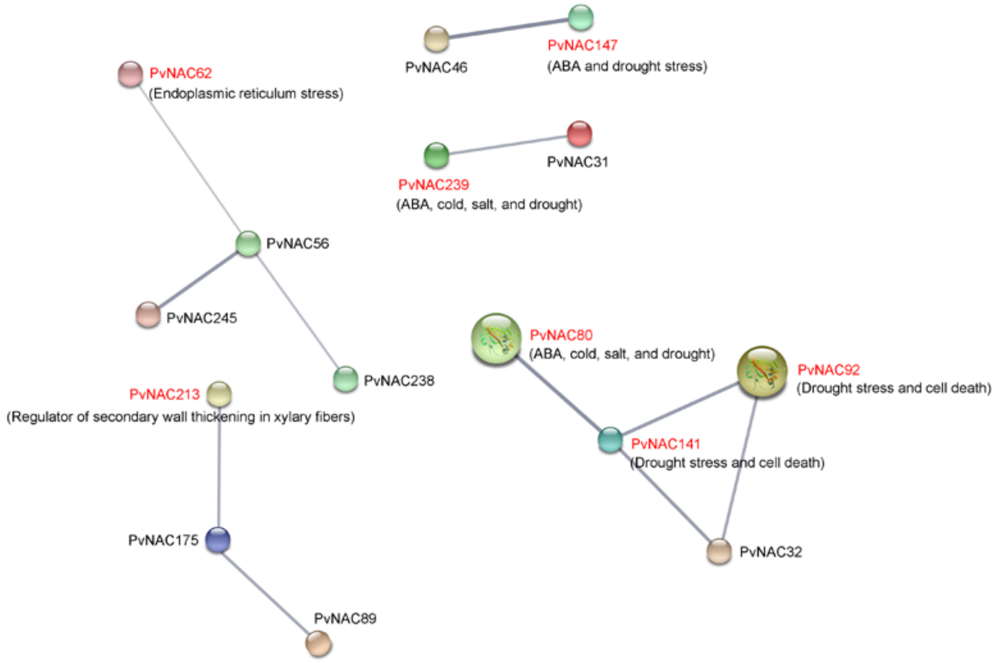

Figure S5 Interaction network analysis of NAC proteins identified in switchgrass according to the orthologs in rice. The line thickness indicated the combined score. The PvNAC proteins with a red color indicated they are potential functional proteins in Table S1, and the predicted functional information was listed below them. This network maybe speculative because it is unknown whether the switchgrass and rice proteins are true orthologues or not, and further studies should be conducted to confirm the exact annotation.

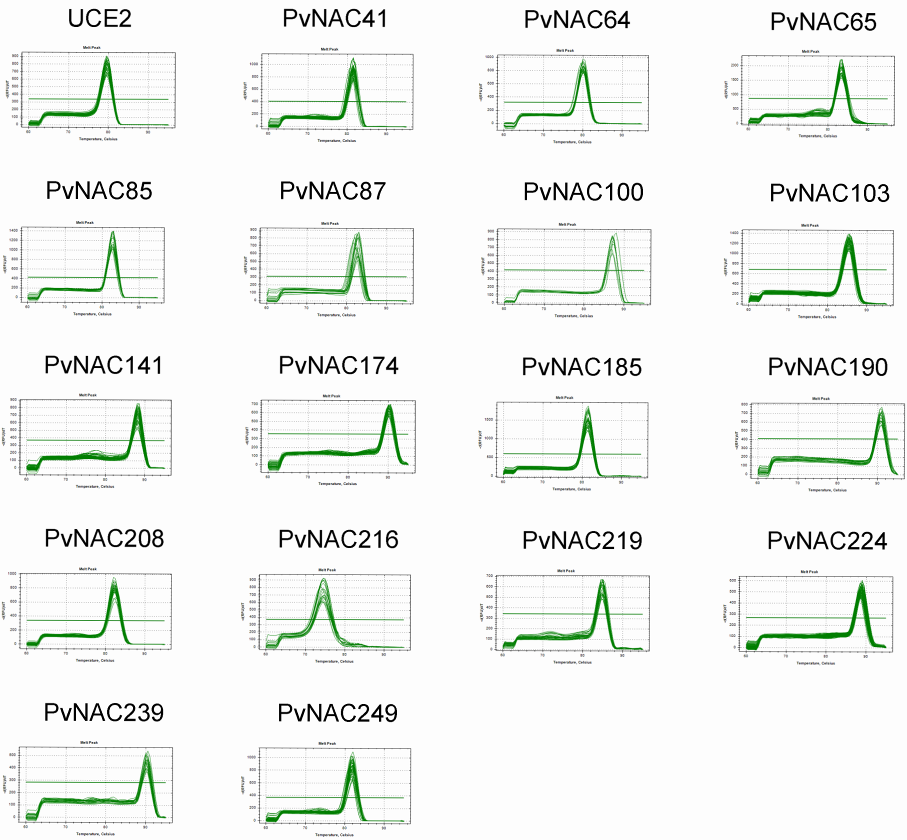
UCE2
PvNAC41
PvNAC64
PvNAC65
PvNAC85
PvNAC87
PvNAC100
PvNAC103
PvNAC141
PvNAC174
PvNAC185
PvNAC190
PvNAC208
PvNAC216
PvNAC219
PvNAC224
PvNAC239
PvNAC249
Melt Peak
Temperature, Celsius

Figure S6 Melting curves of one reference gene (*UCE2*) and 17 *PvNAC* genes showing single peaks in qRT-PCR.

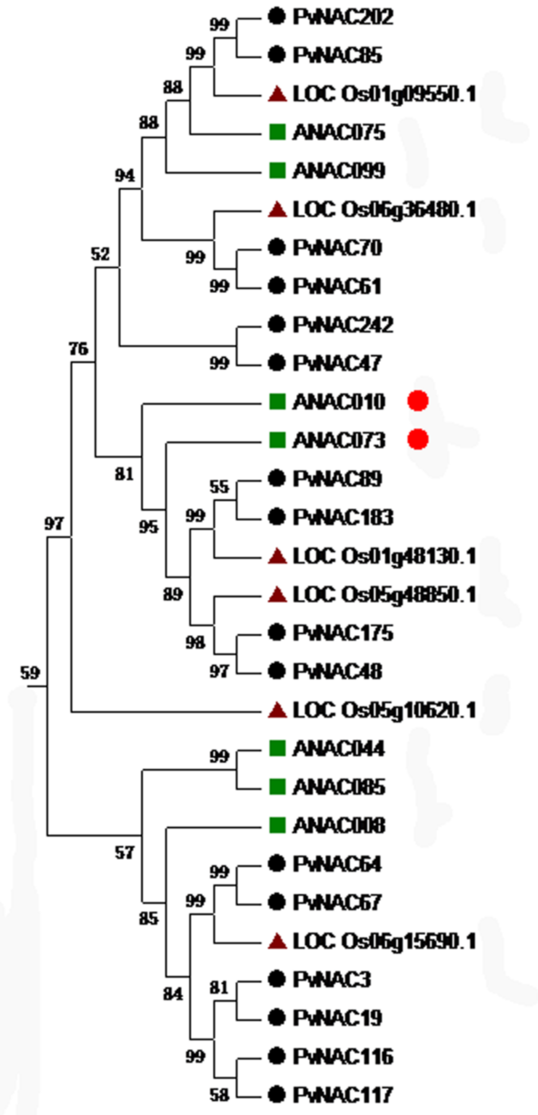

Figure S7 The PvNACs with motif 10 in figure 8 were homologous to ANA010 and ANA073 involved in secondary cell wall thickening in lignified cells.

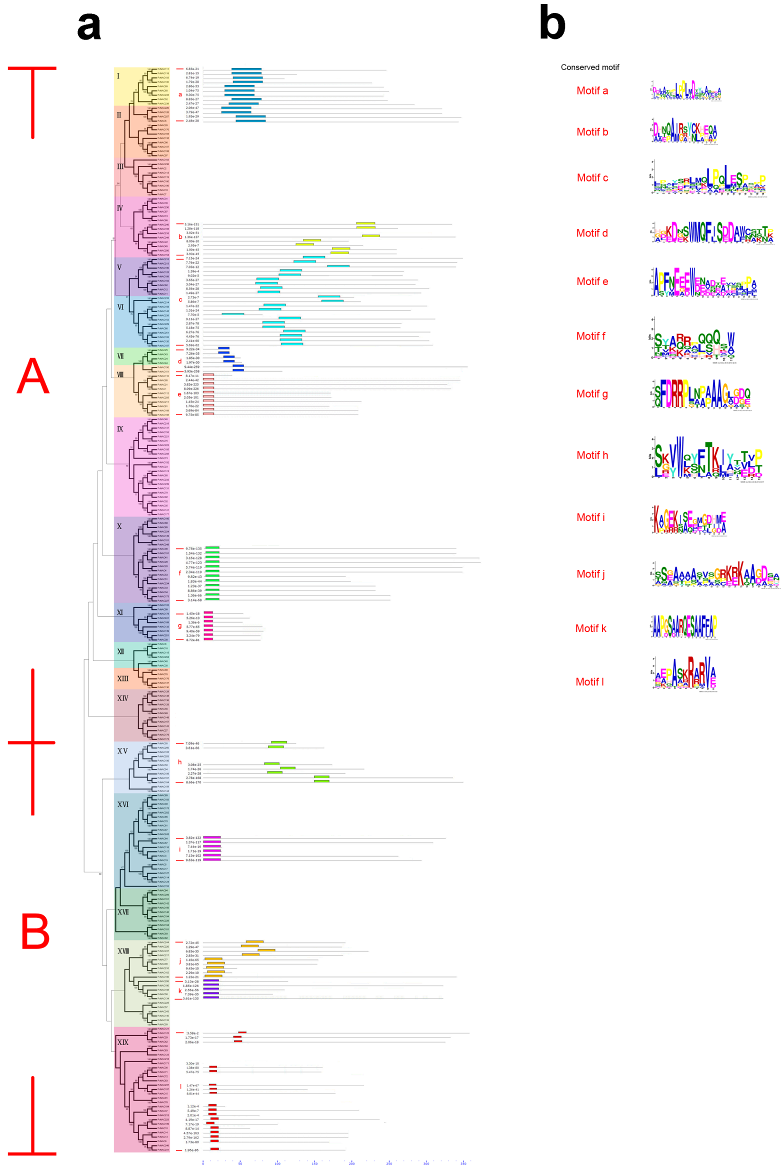

a
b
A
B
Conserved motif
Motif a
Motif b
Motif c
Motif d
Motif e
Motif f
Motif g
Motif h
Motif i
Motif j
Motif k
Motif l
I
II
III
IV
V
VI
VII
VIII
IX
X
XI
XII
XIII
XIV
XV
XVI
XVII
XVIII
XIX

Figure S8 Schematic representation of the C-terminal of PvNACs located TARs (or TRRs) with highly divergent sequences by MEME analysis. a. A total of 12 motifs (a-l) were identified in the TARs. b. The logo of motifs a-l in C-terminal regions.

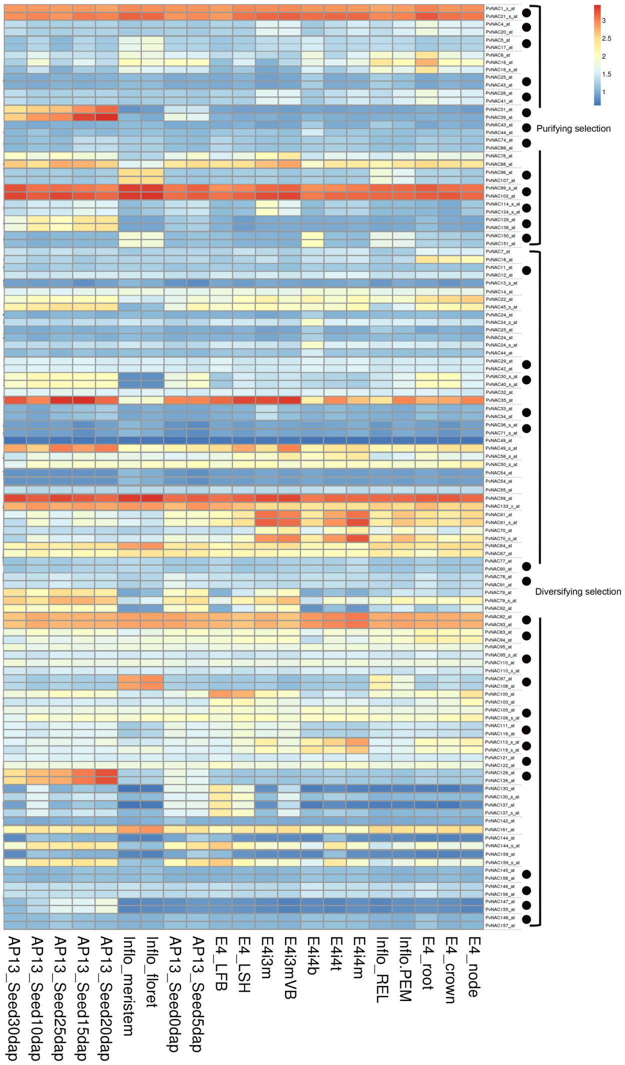

3
2.5
2
1.5
1
Purifying selection
Diversifying selection
PvNAC1_x_at
PvNAC21_s_at
PvNAC4_at
PvNAC20_at
PvNAC5_at
PvNAC17_at
PvNAC8_at
PvNAC16_at
PvNAC16_s_at
PvNAC25_at
PvNAC43_at
PvNAC28_at
PvNAC41_at
PvNAC31_at
PvNAC39_at
PvNAC43_at
PvNAC44_at
PvNAC74_at
PvNAC86_at
PvNAC76_at
PvNAC88_at
PvNAC96_at
PvNAC107_at
PvNAC99_s_at
PvNAC102_at
PvNAC114_s_at
PvNAC124_s_at
PvNAC129_at
PvNAC138_at
PvNAC150_at
PvNAC151_at
PvNAC7_at
PvNAC18_at
PvNAC11_at
PvNAC12_at
PvNAC13_s_at
PvNAC14_at
PvNAC22_at
PvNAC45_s_at
PvNAC24_at
PvNAC24_s_at
PvNAC25_at
PvNAC24_at
PvNAC24_s_at
PvNAC44_at
PvNAC29_at
PvNAC42_at
PvNAC30_s_at
PvNAC40_s_at
PvNAC32_at
PvNAC35_at
PvNAC33_at
PvNAC34_at
PvNAC36_s_at
PvNAC71_s_at
PvNAC49_at
PvNAC49_s_at
PvNAC56_s_at
PvNAC50_s_at
PvNAC54_at
PvNAC54_at
PvNAC55_at
PvNAC59_at
PvNAC133_x_at
PvNAC61_at
PvNAC61_s_at
PvNAC70_at
PvNAC70_s_at
PvNAC64_at
PvNAC67_at
PvNAC77_at
PvNAC90_at
PvNAC78_at
PvNAC91_at
PvNAC79_at
PvNAC79_s_at
PvNAC92_at
PvNAC82_at
PvNAC93_at
PvNAC83_at
PvNAC94_at
PvNAC95_at
PvNAC95_s_at
PvNAC110_at
PvNAC110_s_at
PvNAC97_at
PvNAC108_at
PvNAC100_at
PvNAC103_at
PvNAC105_at
PvNAC106_s_at
PvNAC111_at
PvNAC118_at
PvNAC113_s_at
PvNAC119_s_at
PvNAC121_at
PvNAC122_at
PvNAC128_at
PvNAC136_at
PvNAC130_at
PvNAC130_s_at
PvNAC137_at
PvNAC137_s_at
PvNAC142_at
PvNAC161_at
PvNAC144_at
PvNAC144_s_at
PvNAC159_at
PvNAC159_s_at
PvNAC145_at
PvNAC158_at
PvNAC146_at
PvNAC156_at
PvNAC147_at
PvNAC155_at
PvNAC148_at
PvNAC157_at
AP13_Seed30dap
AP13_Seed10dap
AP13_Seed25dap
AP13_Seed15dap
AP13_Seed20dap
Inflo_meristem
Inflo_floret
AP13_Seed0dap
AP13_Seed5dap
E4_LFB
E4_LSH
E4i3m
E4i3mVB
E4i4b
E4i4t
E4i4m
Inflo_REL
Inflo.PEM
E4_root
E4_crown
E4_node

Figure S9 The tissue level expression between paralogous *PvNAC* pairs under diversifying and purifying selection. The abbreviations of each tissue and developmental stage were derived from PviGEA database. AP13_ Seed0d, AP13_ Seed5d, AP13_ Seed10d, AP13_ Seed15d, AP13_ Seed20d, AP13_ Seed25d, AP13_ Seed30d represent whole flowers at anthesis stage, whole seeds 5 days post fertilization, whole seeds with visible caryopsis, whole seeds at the milk stage, whole seeds at the soft dough stage, whole seeds at the hard dough stage, whole seeds at the physiological maturity stage, respectively. Inflo-meristem: Inflorescence meristem (0.5-3.0 mm). Inflo-floret: Floret of inflorescence when glumes are 10–20 mm. Inflo-REL: Rachis and branch elongation of inflorescence (50–150 mm). Inflo-PEM: Panicle emergence of inflorescence (>200 mm). E4-LFB: Pooled leaf blade from plant. E4-LSH: Pooled leaf sheath. E4i3m: Middle 1/5 fragment of the 3rd internode. E4i3mVB: Vascular bundle isolated from 1/5 fragment of the 3rd internode. E4i4b: Bottom 1/5 fragment of the 4th internode. E4i4t: Top 1/5 fragment of the 4th internode. E4i4m: Middle 1/5 fragment of the 4th internode 4. E4-root: Whole root system. E4-crown: Whole crown. E4-node: Pooled nodes.

Table S1 Plant functional-annotated NAC genes and corresponding orthologs of PvNACs.

Locus name	NAC name	Gene functional annotation	References	Closest PvNAC homolog(s)
Os04g38720.1	OMTN2; OsNAC2	Regulator of the plant height and flowering time; Improve plant structure for higher light-use efficiency and higher yield	Chen et al. 2015 [1]; Mao et al. 2007 [2]	PvNAC126/220 in clade I
Os11g03370.1	ONAC45	Involved in drought and salt regulation	Zheng et al. 2009 [3]	PvNAC249/216/240/195 in clade II
AT1G32770	ANAC012	Regulator of secondary wall thickening in xylary fibers	Jae-Heung et al. 2007 [4]	PvNAC190/213/215 in clade III
Os03g02800.1	ONAC054/RIM1	Component of jasmonic acid (JA) signaling	Yoshii et al. 2009 [5]	PvNAC150/151 in clade IV
Os01g60020.1	OsNAC4; ONAC068	Be related to plant hypersensitive cell death	Takashi et al. 2009 [6]	PvNAC92/79/141/177 in clade V
Os03g60080.1	SNAC1; OsNAC9; OsNAC19	Involved in drought stress regulation	Khandok et al. 2014 [7]	PvNAC92/79/141/177 in clade V
Os05g34830.1	OsNAC52	Involved in ABA and drought stress regulation	Gao et al. 2010 [8]	PvNAC239/232/174/80 in clade V
AT5G63790	ANAC102	Involved in ABA regulation; An important regulator of seed germination under flooding.	Takasaki et al. 2015 [9]; Christianson et al. 2009 [10]	PvNAC239/232/174/80 in clade V
AT5G08790	ANAC081	Involved in ABA regulation	Takasaki et al. 2015 [9]	PvNAC239/232/174/80 in clade V
AT1G01720	ANAC002	Involved in ABA regulation	Takasaki et al. 2015 [9]	PvNAC239/232/174/80 in clade V
AT1G77450	ANAC032	Involved in ABA regulation	Takasaki et al. 2015 [9]	PvNAC239/232/174/80 in clade V
Os01g66120.1	OsNAC6; SNAC2	Involved cold and salt stress regulation	Kazuo et al. 2007 [11]; Hu et al.	PvNAC239/232/174/80 in

			2008 [12]	clade V
Os11g03300.1	OsNAC10; ONAC122	Involved in drought regulation	Sun et al. 2010 [13]; Jin et al. 2010 [14]	PvNAC208/222/185 in clade V
Os12g03040.1	ONAC131	Involved in cold and drought regulation	Sun et al. 2010 [13]	PvNAC208/222/185 in clade V
Os07g48450.1	NAC8	Involved in ABA and drought stress regulation	Nuruzzaman et al. 2012 [15]	PvNAC75/221/147/155/219/46 in clade V
AT2G17040	ANAC036	Involved in the growth of leaf cells.	Kato et al. 2010 [16]	PvNAC166/65/68/224/149 in clade VI
Os08g33910.1	ONAC063	Play an important role in eliciting responses to high-salinity stress	Yokotani et al. 2009 [17]	PvNAC98/191/4/20/41/28 in clade VI
AT3G49530	ANAC062	Involved in endoplasmic reticulum (ER) stress signaling from the plasma membrane to the nucleus and play an important role in regulating UPR downstream gene expression	Yang et al. 2014 [18]	PvNAC99/102 in clade VII
AT4G17980	ANAC071	Be related to ethylene and JA	Asahina et al. 2011 [5]	PvNAC148/157/143 in clade IX
AT1G34190	ANAC017	Play a key role in both biogenic and operational mitochondrial retrograde signalling, and improve plant performance when mitochondrial function is constitutively impaired.	Aken et al. 2016 [19]	PvNAC172/27/179 in clade IX
AT1G32870	ANAC013	Regulator of mitochondrial retrograde regulation (MRR) upon stress.	Clercq et al. 2013 [20]	PvNAC172/27/179 in clade IX

AT4G29230	ANAC075	Involved in flowering-time gene regulation in phloem and/or vascular tissue formation	Fujiwara et al. 2016 [21]	PvNAC202/85 in clade XI
AT2G43000	ANAC042	Key transcription factor involved in previously unknown regulatory mechanisms to induce phytoalexin biosynthesis	Saga et al. 2012 [22]	None
AT5G39610	ANAC092	transcription factor incorporating the environmental and endogenous stimuli into the process of plant lateral root development	He et al. 2005 [23]	None
AT1G52890	ANAC019	Involved in JA and ABA regulation	Qingyun et al. 2008 [24]; Jensen et al. 2010 [25]	None
AT3G15500	ANAC055	Involved in JA and ABA regulation	Qingyun et al. 2008 [24]; Takasaki et al. 2015 [9]	None
AT4G27410	ANAC072	Involved in drought, high salinity, and ABA regulation	Takasaki et al. 2015 [9]	None

Reference

1. Chen, X. *et al.* OsNAC2 encoding a NAC transcription factor that affects plant height through mediating the gibberellic acid pathway in rice. *Plant J Cell Mol Biol*. **82**, 302–14 (2015).

2. Mao, C.Z. *et al.* Overexpression of a NAC‐domain protein promotes shoot branching in rice. *New Phytol*. **176**, 288–98 (2007).

3. Zheng, X.N., Bo, C., Lu, G.J. & Han, B. Overexpression of a NAC transcription factor enhances rice drought and salt tolerance. *Biochem Biophys Res Commun*. **379**, 985-9 (2009).

4. Jae‐Heung, K., Yang, S.H., Park, A.H., Olivier, L. & Han, K.H. ANAC012, a member of the plant-specific NAC transcription factor family, negatively regulates xylary fiber development in *Arabidopsis thaliana*. *Plant J*. **50**, 1035-48 (2007).

5. Asahina, M. *et al.* Spatially selective hormonal control of RAP2.6L and ANAC071 transcription factors involved in tissue reunion in *Arabidopsis*. *PNAS*. **108**, 16128-32 (2011).

6. Kaneda, T. *et al.* The transcription factor OsNAC4 is a key positive regulator of plant hypersensitive cell death. *Embo J*. **28**, 926-36 (2009).

7. Songyikhangsuthor, K. *et al.* Natural variation in the sequence of SNAC1 and its expression level polymorphism in rice germplasms under drought stress. *J Genet Genomics*. **41**, 609-12 (2014).

8. Gao, F. *et al.* OsNAC52 , a rice NAC transcription factor, potentially responds to ABA and confers drought tolerance in transgenic plants. *Plant Cell Tissue Organ Cult*. **100**, 255-62 (2009).

9. Takasaki, H. *et al.* SNAC-As, stress-responsive NAC transcription factors, mediate ABA-inducible leaf senescence. *Plant J Cell Mol Biol*. **84**, 1114-23 (2015).

10. Christianson, J.A. & Dennis, E.S. The low-oxygen-induced NAC domain transcription factor ANAC102 affects viability of *Arabidopsis* seeds following low-oxygen treatment. *Plant Physiol*. **149**, 1724-38 (2009).
11. Nakashima, K. *et al.* Functional analysis of a NAC-type transcription factor OsNAC6 involved in abiotic and biotic stress-responsive gene expression in rice. *Plant J*. **51**, 617-30 (2007).
12. Hu, H.H. *et al.* Characterization of transcription factor gene SNAC2 conferring cold and salt tolerance in rice. *Plant Mol Biol*. **67**, 169-81 (2008).
13. Sun, L. *et al.* Functions of rice NAC transcriptional factors, ONAC122 and ONAC131, in defense responses against *Magnaporthe grisea*. *Plant Mol Biol*. **81**, 41-56 (2013).
14. Jin, S.J. *et al.* Root-specific expression of OsNAC10 improves drought tolerance and grain yield in rice under field drought conditions. *Plant Physiol*. **153**, 185-97 (2010).
15. Nuruzzaman, M. *et al.* Comprehensive gene expression analysis of the NAC gene family under normal growth conditions, hormone treatment, and drought stress conditions in rice using near-isogenic lines (NILs) generated from crossing Aday Selection (drought tolerant) and IR64. *Mol Genet Genomics*. **287**, 389-410 (2012).
16. Kato, H., Motomura, T., Komeda, Y., Saito, T. & Kato, A. Overexpression of the NAC transcription factor family gene ANAC036 results in a dwarf phenotype in *Arabidopsis thaliana*. *J Plant Physiol*. **167**, 571-7 (2010).
17. Yokotani, N. *et al.* Tolerance to various environmental stresses conferred by the salt-responsive rice gene ONAC063 in transgenic *Arabidopsis*. *Planta*. **229**, 1065-75 (2009).
18. Yang, Z.T. *et al.* A plasma membrane-tethered transcription factor, NAC062/ANAC062/NTL6, mediates the unfolded protein response in *Arabidopsis*. *Plant J Cell Mol Biol*. **79**, 1033-43 (2014).
19. Van Aken, O., Ford, E., Lister, R., Huang, S. & Millar, A.H. Retrograde signalling caused by heritable mitochondrial dysfunction is partially mediated by ANAC017 and improves plant performance. *Plant J*. doi: 10.1111/tpj.13276 (2016).
20. Clercq, I.D. *et al.* The membrane-bound NAC transcription factor ANAC013 functions in mitochondrial retrograde regulation of the oxidative stress response in *Arabidopsis*. *Plant Cell*. **25**, 3472-90 (2013).
21. Fujiwara, S. & Mitsuda, N. ANAC075, a putative regulator of VASCULAR-RELATED NAC-DOMAIN7, is a repressor of flowering. *Plant Biotechnol*. doi: 10.5511/plantbiotechnology.16.0215b (2016).
22. Saga, H. *et al.* Identification and characterization of ANAC042, a transcription factor family gene involved in the regulation of camalexin biosynthesis in *Arabidopsis*. *Mol Plant Microbe Interact*. **25**, 684-96 (2012).
23. †, X.J.H. *et al.* AtNAC2, a transcription factor downstream of ethylene and auxin signaling pathways, is involved in salt stress response and lateral root development. *Plant J*. **44**, 903-16 (2006).
24. Bu, Q.Y. *et al.* Role of the *Arabidopsis thaliana* NAC transcription factors ANAC019 and ANAC055 in regulating jasmonic acid-signaled defense responses. *Cell Res*. **18**, 756-67 (2008).
25. Jensen, M.K. *et al.* The *Arabidopsis thaliana* NAC transcription factor family: structure-function relationships and determinants of ANAC019 stress signalling. *Biochem J*. **426**, 183-96 (2009).

Table S2 Diversifying and purifying selection of PvNACs.

	Chromosomal locations	Ks	Ka	Ka/Ks	Evolutionary Selection	Duplication type
PvNAC1/21	Chr01b/01a	0.670	0.638	0.953	Purifying	Homeologous
PvNAC4/20	Chr01b/01a	0.067	0.015	0.220	Purifying	Homeologous
PvNAC5/17	Chr01a/01b	2.008	1.767	0.880	Purifying	Homeologous
PvNAC7/18	Chr01b/01a	0.447	0.636	1.423	Diversifying	Homeologous
PvNAC8/16	Chr01a/01b	0.125	0.044	0.348	Purifying	Homeologous
PvNAC11/12	Chr01b/01a	0.432	0.485	1.122	Diversifying	Homeologous
PvNAC13/14	Chr01b/01b	1.223	6.145	5.025	Diversifying	Homeologous
PvNAC22/45	Chr02a/02b	0.963	3.567	3.705	Diversifying	Homeologous
PvNAC24/44	Chr02a/02b	0.907	2.686	2.962	Diversifying	Homeologous
PvNAC25/43	Chr02a/02b	0.059	0.012	0.202	Purifying	Homeologous
PvNAC28/41	Chr02b/02a	1.328	1.261	0.950	Purifying	Homeologous
PvNAC29/42	Chr02a/02b	0.861	2.003	2.325	Diversifying	Homeologous
PvNAC30/40	Chr02b/02a	1.175	2.559	2.177	Diversifying	Homeologous
PvNAC31/39	Chr02a/02b	1.701	0.000	0.000	Purifying	Homeologous
PvNAC32/35	Chr02a/02b	0.560	4.367	7.802	Diversifying	Homeologous
PvNAC33/34	Chr02a/02b	1.169	3.506	3.000	Diversifying	Homeologous
PvNAC36/71	Chr02b/04b	1.206	3.413	2.830	Diversifying	Paralogous
PvNAC49/56	Chr03b/03a	0.389	0.624	1.604	Diversifying	Homeologous
PvNAC50/54	Chr03a/03b	1.177	2.660	2.260	Diversifying	Homeologous
PvNAC59/133	Chr08a/03b	0.590	0.969	1.641	Diversifying	Paralogous
PvNAC61/70	Chr04b/04a	0.568	0.626	1.102	Diversifying	Homeologous
PvNAC64/67	Chr04a/04b	2.120	2.356	1.111	Diversifying	Homeologous
PvNAC74/86	Chr05a/05b	4.431	2.835	0.640	Purifying	Homeologous
PvNAC76/88	Chr05b/05a	0.457	0.317	0.692	Purifying	Homeologous

PvNAC77/90	Chr05a/05b	0.738	1.819	2.466	Diversifying	Homeologous
PvNAC78/91	Chr05b/05a	0.934	2.520	2.699	Diversifying	Homeologous
PvNAC79/92	Chr05a/05b	0.552	1.843	3.338	Diversifying	Homeologous
PvNAC82/93	Chr05b/05a	0.360	0.611	1.698	Diversifying	Homeologous
PvNAC83/94	Chr05b/05a	0.687	1.396	2.033	Diversifying	Homeologous
PvNAC95/110	Chr06b/06a	0.850	1.913	2.250	Diversifying	Homeologous
PvNAC96/107	Chr06a/06b	0.788	0.000	0.000	Purifying	Homeologous
PvNAC97/108	Chr06b/06a	0.817	3.836	4.695	Diversifying	Homeologous
PvNAC99/102	Chr06b/06a	0.711	0.699	0.983	Purifying	Homeologous
PvNAC100/103	Chr06b/06a	1.100	3.028	2.754	Diversifying	Homeologous
PvNAC111/118	Chr07a/07b	0.393	0.468	1.190	Diversifying	Homeologous
PvNAC113/119	Chr07a/07b	0.427	0.656	1.536	Diversifying	Homeologous
PvNAC114/124	Chr07a/07b	0.121	0.092	0.757	Purifying	Homeologous
PvNAC121/122	Chr07b/07b	0.437	0.927	2.122	Diversifying	Homeologous
PvNAC128/136	Chr08b/08a	2.518	2.700	1.072	Diversifying	Homeologous
PvNAC129/138	Chr08a/08b	0.328	0.259	0.792	Purifying	Homeologous
PvNAC130/137	Chr08b/08a	1.179	1.257	1.067	Diversifying	Homeologous
PvNAC142/161	Chr09b/09a	0.880	1.876	2.132	Diversifying	Homeologous
PvNAC144/159	Chr09b/09a	3.002	3.384	1.127	Diversifying	Homeologous
PvNAC145/158	Chr09b/09a	0.473	0.809	1.709	Diversifying	Homeologous
PvNAC146/156	Chr09b/09a	1.075	2.353	2.189	Diversifying	Homeologous
PvNAC147/155	Chr09a/09b	0.298	0.389	1.307	Diversifying	Homeologous
PvNAC148/157	Chr09a/09b	0.564	0.862	1.529	Diversifying	Homeologous
PvNAC150/151	Chr09a/09b	0.577	0.499	0.864	Purifying	Homeologous
PvNAC24/25	Chr02a/Chr02a	2.632	3.792	1.440	Diversifying	Tandem
PvNAC43/44	Chr02b/Chr02b	2.069	0.685	0.331	Purifying	Tandem

PvNAC54/55	Chr03b/Chr03b	2.262	2.723	1.203	Diversifying	Tandem
PvNAC105/106	Chr06b/Chr06b	2.632	3.792	1.440	Diversifying	Tandem

Table S3 Local blast between NAC genes of Shen's study (2009) and our study.

	NAC genes in Shen's study	NAC genes in our study
1	PvNAC032	PvNAC89
2	PvNAC055	PvNAC190
3	PvNAC033	PvNAC183
4	PvNAC046	PvNAC16
5	PvNAC061	PvNAC215
6	PvNAC062	PvNAC215
7	PvNAC066	PvNAC85
8	PvNAC068	PvNAC152
9	PvNAC101	PvNAC213
10	PvNAC102	PvNAC16

Reference

Shen, H., Yin, Y.B., Chen, F., Xu, Y. & Dixon, R.A. A Bioinformatic analysis of NAC genes for plant cell wall development in relation to lignocellulosic bioenergy production. *Bioenerg Res*. **2**, 217-32 (2009).

Table S4 The interaction relationship for PvNAC proteins and their orthologous rice NAC proteins.

Node 1 PvNACs	Node 1 rice NAC proteins	Node 2 PvNACs	Node 2 rice NAC proteins	Score
PvNAC92	LOC_Os01g60020.1	PvNAC32	LOC_Os07g12340.1	0.606
PvNAC141	LOC_Os03g60080.1	PvNAC92	LOC_Os01g60020.1	0.687
PvNAC62	LOC_Os06g33940.1	PvNAC56	LOC_Os12g29330.1	0.414
PvNAC245	LOC_Os06g46270.1	PvNAC56	LOC_Os12g29330.1	0.725
PvNAC238	LOC_Os12g41680.1	PvNAC56	LOC_Os12g29330.1	0.479
PvNAC175	LOC_Os05g48850.1	PvNAC213	LOC_Os08g02300.1	0.582
PvNAC89	LOC_Os01g48130.2	PvNAC175	LOC_Os05g48850.1	0.601
PvNAC46	LOC_Os07g48450.1	PvNAC147	LOC_Os03g21060.1	0.814
PvNAC239	LOC_Os02g12310.1	PvNAC31	LOC_Os01g29840.1	0.582
PvNAC80	LOC_Os01g66120.1	PvNAC141	LOC_Os03g60080.1	0.767
PvNAC32	LOC_Os07g12340.1	PvNAC141	LOC_Os03g60080.1	0.678

Table S5 The primers of genes used to be validated.

Gene	Forward primer	Reverse primer	Amplification efficiency (%)	
PvNAC41	CTATCTCAAGCGAAAGATTCAGC	ATATGGGCCTATCTGTTCCTGTT	95.63	Stress-related genes
PvNAC141	AGTCGGAGATCGTGGACAAC	GAAGAGCGACGAGTAGAAGTCCT	92.15	
PvNAC174	GTACGGCGAGAAGGAGTGGTACT	GTACTCGTGCATGATCCAGTTG	95.74	
PvNAC185	AACCTGAACCACCGCTAA	ACTGCTGCTACCATCCAA	97.43	
PvNAC208	TCTCCTCAACACCATCGACTACT	CATAGTCATCACTCGCTTCCTCT	98.22	
PvNAC216	AGTTTCTGAACCCTAGCTTCGAC	CTTGAAGATCTCCCTGTCCTTG	88.39	
PvNAC219	GCTTCTTCATTCTTGTCGATCTC	GACATCATCATCTCCATCTCCAT	91.28	
PvNAC239	GGAGGAGGAATCAATCAAGAAAG	AGGGGTCGAACTTGTAGAGGTC	89.90	
PvNAC249	CAACAAACAACAGTGGCAGTTAC	TCACCGTAGTTGTTGCCTAACTT	93.68	
PvNAC65	CCTCTGCACCGTTCGTCAC	GTAGCCTTCCGTTGTCCTTCTC	102.87	
PvNAC103	AGGAGGACTGGGTGCTATGC	CAGTGTTGGTGTCTGTGAGGG	98.88	
PvNAC224	TGGAAGCCAAGCAGGAAGC	CGCCAAGGGAGACAAGAAA	96.58	
PvNAC64	ACAATCTGGAGCATAACC	GAATCACCATCCCACAAC	90.33	Tissue-specific genes
PvNAC87	AAGGATGGCTACTGGAAG	AAGGAGGTTGAACTGGATG	88.67	
PvNAC239	GGAGGAGGAATCAATCAAGAAAG	AGGGGTCGAACTTGTAGAGGTC	91.20	
PvNAC85	ATGTGCCAGCAATCCTTC	GGCGGCCAGTATGGGTAA	97.28	
PvNAC190	CAGCAGCAAGCACCACCAGG	CCGATCCAGCGTGTCCCAGT	90.89	
PvNAC100	GCAGAACAACCAACCCAAAG	AGAAGGTCACCAAGGCTCACT	91.93	
UCE2	TATATGACGGAGGCTACT	CAGGTGGATGAAGAATAGA	95.34	Reference gene
